# Bone health, obesity, and multimorbidity risk among teachers in under-resourced South African public primary schools: results from a cross-sectional DXA study

**DOI:** 10.3389/fpubh.2026.1794604

**Published:** 2026-10-09

**Authors:** Nandi Joubert, Larissa Adams, Harald Seelig, Emma Cora Gardiner, Riaan van de Venter, Jan Degen, Danielle Dolley, Ivan Müller, Siphesihle Nqweniso, Nicole Probst-Hensch, Peter Steinmann, Uwe Pühse, Rosa du Randt, Markus Gerber, Jürg Utzinger, Cheryl Walter

**Affiliations:** 1 Department of Human Movement Science, Nelson Mandela University, Gqeberha, South Africa; 2 Swiss Tropical and Public Health Institute, Allschwil, Switzerland; 3 University of Basel, Basel, Switzerland; 4 Department of Sport, Exercise and Health, University of Basel, Basel, Switzerland; 5 Department of Internal Medicine, Livingstone Hospital, Gqeberha, South Africa; 6 Department of Radiography, Nelson Mandela University, Gqeberha, South Africa; 7 Department of Human Movement Science, University of Fort Hare, Alice, South Africa

**Keywords:** bone density, dual-energy X-ray absorptiometry, metabolic syndrome, obesity, South Africa, body composition, teachers, multimorbidity

## Abstract

**Introduction:**

In low- and middle-income countries, adverse body composition risk is increasingly driven by structural determinants, including rapid urbanisation, nutrition transitions, and reduced opportunities for physical activity. Quantifying and interpreting bone, fat and lean mass risks require appropriate measurement and context-sensitive thresholds to detect subclinical vulnerability in under-resourced settings. Few studies, particularly from Africa, have quantified how these risks intersect and cluster with cardiometabolic and socio-demographic determinants. The purpose of this study was to characterise body composition profiles, estimate the prevalence and co-occurrence of risk phenotypes and examine their distribution across health determinants.

**Methods:**

The study was carried out in under-resourced public schools in Gqeberha, Eastern Cape, South Africa, as part of the *KaziBantu* project (ISRCTN18485542). A school-based workplace health intervention was conducted, using cross-sectional assessments. We employed dual-energy X-ray absorptiometry (DXA), assessed metabolic indicators, behavioural indicators (alcohol and tobacco use, device-measured physical activity, handgrip strength), and obtained demographic and medical data.

**Results:**

Among 136 apparently healthy teachers (85% women; age 21–72 years; mean = 47 years; standard deviation = 11 years), body composition risk was prevalent: fat mass risk 91.8% (*n* = 123/134; 95%CI 85.8–95.8%), lean mass risk 15.7% (*n* = 21/134; 95%CI 10–23%) and bone mass risk 29.9% (*n* = 40/134; 95%CI 22.3–38.4%). The most frequent combined phenotype was bone-fat risk (27.6%; *n* = 37; 95%CI 20.2–36%), while 6% (*n* = 8; 95%CI 2.6–11.4%) exhibited all three risks concurrently. Metabolic syndrome criteria were met in 53% (*n* = 70; 95%CI 44.2–61.8%) of teachers, and adverse risk phenotypes were associated with elevated multimorbidity and poorer metabolic health.

**Conclusion:**

This is the first study from South Africa’s Eastern Cape to report DXA-derived body composition profiles. Body composition risks and multimorbidity were prevalent, with likely substantial impacts on quality of life. Protecting teachers’ health will require multi-level strategies, including workplace-based screening with low-cost tools (e.g., bioelectrical impedance), culturally responsive prevention such as targeted weight-bearing and resistance activity, and action on structural inequities, such as more equitable resourcing across schools to reduce occupational strain and stronger pathways that turn teachers’ existing health coverage into timely screening and treatment. These strategies aim to safeguard well-being and sustain teachers’ pivotal role in educating future generations.

## 1 Introduction

Obesity is one of the most prevalent disorders and a major contributor to non-communicable diseases (NCDs) (1). NCDs account for nearly three-quarters of all deaths globally and in the World Health Organization (WHO) African Region, more than 2.8 million NCD-related deaths were recorded in 2019 (2). Low- and middle-income countries (LMICs) bear a high NCD burden, accounting for 77% of all NCD deaths and the highest rates of premature mortality (3). Obesity typically clusters with cardiometabolic, musculoskeletal, endocrine, neurological and psychosocial health, reducing quality of life and hindering social and economic development (1). Through ectopic fat deposition, chronic low-grade inflammation, adipokine dysregulation and endocrine disturbances, excess adiposity also disrupts bone remodelling and suppresses muscle protein synthesis and function, increasing fracture risk, frailty and healthcare utilisation (4). The co-occurrence of excess adiposity with low bone and/or muscle is further linked to hypertension, dyslipidaemia, insulin resistance and hyperglycaemia (5). Assessing body composition across bone, fat and lean mass therefore provides independent and complementary insights into musculoskeletal health, cardiometabolic risk, functional capacity and well-being. To interpret these interrelated risks, appropriate measurement and context-sensitive definitions of risk are essential.

Obesity is commonly defined using body mass index (BMI), which can misestimate adiposity because it reflects weight for height and does not distinguish tissue types (6). Anthropometric estimates (e.g., skinfolds) and direct measures (e.g., dual-energy X-ray absorptiometry [DXA]) provide a more valid assessment of adipose tissue and fat-mass risk. In terms of lean mass, sarcopenia refers to muscle mass and strength decline with functional impairment, which is most evident in older adults. The latest criteria from the European Working Group on Sarcopenia in Older People (EWGSOP2) identify low muscle strength as the primary criterion for sarcopenia, confirm diagnosis with low muscle quantity or quality, and grade severity by poor physical performance (7, 8). For population surveillance, especially in working-age adults, strict EWGSOP2 case definitions can be too specific as they rely on performance tests that are not always feasible and are derived from cut-points largely from older European populations. To detect earlier, subclinical vulnerabilities, more liberal constructs of lean mass risk are warranted. Identifying subclinical vulnerabilities is intended to complement clinical diagnosis and is consistent with EWGSOP2’s emphasis on earlier detection and intervention (8). Osteoporosis, a skeletal disorder characterised by reduced bone strength, low bone mineral density (BMD) and microarchitectural deterioration, increases the risk of fragility fractures, disability and premature mortality (9). Clinical thresholds defining osteoporosis are usually applied to adults aged above 50 years and designed for individual diagnosis and treatment, although large population-based studies have shown that bone health impairments can be present before the age of 50 years, especially in postmenopausal women (10). Obesity, sarcopenia and osteoporosis are global public health concerns, with increasing prevalence, particularly in LMICs, as population growth and ageing intersect with urbanisation, nutritional transitions, increasing physical inactivity and limited access to screening and healthcare.

In sub-Saharan Africa, obesity is projected to rise sharply, with a > 2.5-fold increase by 2050, likely intensifying health inequities shaped by socio-economic context, gender, race and ethnicity (11). In LMICs, the growing obesity burden coexists with persistent child undernutrition and infectious diseases, straining healthcare systems and exerting considerable economic costs (US$ 3.19 billion in low-income countries in 2019) (12, 13). Sarcopenia estimates vary by criteria and setting, but a meta-analysis from Africa suggests a weighted prevalence of 25.7%, with risk factors including older age, woman, malnutrition, lower educational attainment and excess adiposity (14, 15). Globally, osteoporosis affects an estimated 18.3% of adults, although African DXA data remain scarce (10). South African DXA studies, limited to small and population-specific cohorts, have reported race-related differences in BMD and stronger associations of lean than fat mass with BMD (16). Studies have also reported human immunodeficiency virus (HIV)/antiretroviral therapy (ART)-related changes in body composition and BMD declines (17–19) and links between DXA-derived adiposity and cardiometabolic risk with longitudinal gains in fat, and losses in lean mass (20). To date, these studies have been confined to Gauteng, Western Cape, KwaZulu-Natal and the North West provinces in South Africa, with none including participants from the Eastern Cape. Moreover, occupational patterns characterised by low-impact loading and prolonged sedentary work, common in teaching, may compound bone, fat and muscle risk, particularly in under-resourced settings (21). Taken together, the projected rise in adiposity, substantial sarcopenia and osteoporosis burdens, occupational risk profiles and regional data gaps underscore the need for context-specific, DXA-based assessments that capture not only single-domain risks but combined risk phenotypes and their links to socio-demographic factors and cardiometabolic health.

The purpose of this study was therefore three-fold. First, to characterise bone health, fat mass and lean mass profiles in an under-researched population of teachers working in low-income areas in the Eastern Cape province of South Africa. Second, to determine the prevalence of bone mass, fat mass, and lean mass risk phenotypes and to report on their co-occurrence (bone-fat risk, fat-lean risk, bone-lean risk and bone-fat-lean risk). Third, to describe how these phenotypes are distributed across key demographic, anthropometric, metabolic, behavioural and medical indicators, thereby providing a comprehensive profile of body composition risk in this occupational group.

## 2 Materials and methods

### 2.1 Study design

This study forms part of the ‘*KaziBantu*: Healthy Schools for Healthy Communities’ project, a cluster-randomised controlled trial conducted from 2019 to 2022. The trial aimed to evaluate the impact of a comprehensive school-based health promotion programme. Detailed information about the study has been published previously (22). In brief, the *KaziBantu* project comprised two components: *KaziKidz*, which targeted the promotion of health and physical activity among schoolchildren, and *KaziHealth*, which used an embedded exploratory design to address teachers’ physical and mental well-being through a workplace intervention. The cross-sectional data used in this manuscript were derived from the baseline assessments of *KaziHealth*.

### 2.2 Setting and participants

The *KaziBantu* project took place in 8 under-resourced public primary schools located in two peri-urban areas of Gqeberha (formerly Port Elizabeth) within the Nelson Mandela Bay Municipality in South Africa’s Eastern Cape province. The study setting is characterised by limited access to essential services and infrastructure, coupled with significant socioeconomic challenges. Principals of under-resourced public primary schools in the Nelson Mandela Bay Municipality were invited to information-sharing sessions held at the Eastern Cape Department of Education in October 2018. Of approximately 349 eligible schools in the municipality, representatives from around 200 attended, and 64 schools expressed interest. From these, 8 schools representative of typical under-resourced primary schools were purposively selected on the basis of: (i) geographical location and representation of the target communities; (ii) language of instruction (isiXhosa, Afrikaans, or English); and (iii) commitment by the school to support the project activities. Full details of the school selection and sampling procedure are reported in the study protocol (22). At the individual level, all teachers employed at the participating schools (*n* = 206) were invited to take part in the *KaziHealth* workplace health intervention. Teachers were enrolled if they (i) were willing to participate, (ii) provided written informed consent, and (iii) were not participating in any other clinical trial during the study period (22). The cross-sectional analyses reported here were restricted to teachers who completed a DXA assessment; teachers with contraindications to DXA (e.g., pregnancy or metal artefacts) were screened at the time of scanning and were therefore not represented in the analytic sample, rather than excluded at enrolment. No formal sample-size calculation was undertaken because this was a descriptive, cross-sectional analysis with the aim of estimation rather than hypothesis testing.

#### 2.2.1 Patient and public involvement

Teachers, principals, and school governing bodies were engaged in an advisory capacity to optimise study design, data collection, logistics and minimise disruption to teaching. One school hosted pilot testing of the assessment tools and visit flow, which led to a streamlined schedule aligned with school timetables. Travel costs and refreshments were provided, and translation support was available if needed. Study findings were returned in one-to-one consultations with a healthcare professional, and aggregated summaries were presented to each school in dedicated information sessions.

### 2.3 Assessments

#### 2.3.1 Demographics

All data assessments took place between May and July 2019. Demographic data were self-reported and included date of birth (age in years as of 2019), biological sex and race. Collecting information on race was considered important given that many participants belonged to groups historically marginalised under South Africa’s apartheid and colonial systems and this information can provide insights into health disparities. Recognising race as a social construct and not a biological determinant, observed differences are seen in light of the broader context of socioeconomic and structural inequalities. Participants reported their race using South Africa’s official categories (i.e., Black, Coloured and Indian, and White). Additional data included educational attainment (diploma, university degree, or postgraduate degree) and average monthly household income: lower (≤R 20,000), middle (R 20,001–40,000) or higher (>R 40,000), consistent with teachers’ salary scales in South Africa.

#### 2.3.2 Body composition

##### 2.3.2.1 Anthropometric indicators

Height and weight were measured by trained personnel following the standardised procedures of the WHO STEPwise approach to surveillance (STEPS) (23), with participants in light clothing and without shoes. Body weight was measured to the nearest 0.1 kg using a bioelectrical impedance scale (Tanita MC-580, Tanita Corp.; Tokyo, Japan), positioned on a firm, level surface. Body height was measured to the nearest 0.1 cm using a Seca stadiometer (Surgical SA; Johannesburg, South Africa), with the participant’s head positioned in the Frankfort plane.

##### 2.3.2.2 Dual-energy X-ray absorptiometry scan

Bone, fat, and lean mass were measured by DXA. Scans were performed by 3 Health Professions Council of South Africa (HPCSA)-registered diagnostic radiographers (each >15 years’ experience) on a Hologic Discovery QDR 4500A with APEX system software v4.0.2 and analysis software v13.4.2 (Hologic Inc., Bedford, MA, USA). Before each scan, daily quality control checked radiation output and the absorption coefficient were measured; the coefficient of variation (CV) for total BMD across scans was 1.0% (24). Radiation dose was recorded from the machine’s dose-area product (DAP) and stored on site, in line with the South African Health Products Regulatory Authority (SAHPRA) guideline for bone densitometers (25). Contraindications (e.g., metal artefacts, pregnancy) were screened and participants removed metal items and changed into gowns for analysis.

Four scans per participant (femoral neck, total hip, lumbar spine, whole body) followed standard protocols (24). For each site, area (cm^2^) and bone mineral content (BMC; g) were assessed; whole-body outputs included body mass, total bone mass, fat mass, and lean mass. Fat mass was expressed as percentage body fat and partitioned into android and gynoid regions; lean mass was partitioned by limbs.

DXA software generated both T- and Z-scores (score definitions and cut-offs in Table 1) for all participants using manufacturer-provided National Health and Nutrition Examination Survey (NHANES) reference data (sex-matched; T relative to the young-adult mean; Z relative to the age-matched mean). Bone-mass risk was classified using the lowest site value (femoral neck, total hip, lumbar spine, or whole body) and age-specific rules were applied. Adults >50 years (*n* = 69) were classified by T-scores (Z-scores summarised descriptively only), whereas adults ≤50 years (*n* = 67) were classified by Z-scores alone and diagnostic labels (osteopenia/osteoporosis) were not applied.

**Table 1:** Derived body composition composite measures with formulas and classification criteria.

Index or ratio	Formula	Units	Classification		
Bone mass indicators					
Bone mineral density (BMD) (9)	$\mathrm{BMD}=\frac{\text{bone mineral content}\;(g)}{\text{bone area}\;(cm^{2})}$	g/cm^2^	–		
T-score >50 years (9)	$T-\text{score}=\frac{\text{patien}t^{\prime }s\;\mathrm{BMD}-\text{mean}\;\mathrm{BMD}\;\text{of young adult reference}\;}{\text{standard deviation of young adult reference}\;}$	-	Normal	≥ − 1.0	
			Osteopenia	−2.5– −1.0	
			Osteoporosis	≤ − 2.5	
Z-score (9)	$Z-\text{score}=\frac{\text{patien}t^{\prime }s\;\mathrm{BMD}\;\text{of}\;\mathrm{age}-\text{matched reference}}{\text{standard deviation of}\;\mathrm{age}-\text{matched reference}}$	-	Expected range for age	≥ − 2.0	
			Below expected range for age	<−2.0	
Fat mass indicators					
Fat mass index (FMI) (54)	$\mathrm{FMI}=\frac{\mathrm{fat}\;\text{mass}\;(\mathrm{kg})}{\text{height}\;{\left(m\right)}^{2}}$	kg/m^2^		Women	Men
			Fat deficit	<5	<3
			Normal	5–9	3–6
			Excess fat	>9–13	>6–9
			Obese class I	>13–17	>9–12
			Obese class II	>17–21	>12–15
			Obese class III	>21	>15
Android/gynoid ratio (A/G ratio) (55)	$A/G\;\text{ratio}=\frac{\text{android}\;\mathrm{fat}\;\text{mass}\;(g)}{\text{gynoid}\;\mathrm{fat}\;\text{mass}\;(g)}$	-		Women	Men
			Low risk	≤0.8	≤1.0
			High risk	>0.8	>1.0
Lean mass indicators					
Total lean mass index (TLMI) (54)	$\text{TLMI}=\frac{\text{total lean mass}\;(\mathrm{kg})}{\text{height}\;{\left(m\right)}^{2}}$	kg/m^2^		Women	Men
			Low	<14.0	<16.5
			Normal	14.0–17.5	16.5–19.5
			High	>17.5	>19.5
Appendicular lean mass index (ALMI) (28)	$\text{ALMI}=\frac{\text{appendicular lean mass}\;(\mathrm{kg})}{\text{height}\;{\left(m\right)}^{2}}$	kg/m^2^		Women	Men
			Low	<5.5	<7.0
			Normal	≥5.5	≥7.0
Anthropometric indicators					
Body mass index (BMI) (31)	$\mathrm{BMI}=\frac{\text{weight}\;(\mathrm{kg})}{{\left[\text{height}\;(m)\right]}^{2}}$	kg/m^2^	Underweight	<18.5	
			Normal	18.5–24.9	
			Overweight	25–29.9	
			Obesity class I	30–34.9	
			Obesity class II	35–39.9	
			Obesity class III	≥40	
Fat-to-muscle ratio (FMR) (56)	$\mathrm{FMR}=\frac{\text{total}\;\mathrm{fat}\;\text{mass}\;(\mathrm{kg})}{\text{total lean mass}\;(\mathrm{kg})}$	-		Women	Men
			Normal	<0.50	<0.35
			Elevated risk	≥0.50	≥0.35

##### 2.3.2.3 Composite measures

In addition to direct anthropometric and DXA-derived measures, a range of calculated indices and ratios were used to characterise bone-, fat- and lean mass. These variables were derived using standard formulas and are summarised in Table 1.

##### 2.3.2.4 Phenotype classifications

Risk phenotype classifications were based on the following criteria: bone mass risk was defined as a T-score <−1.0 in the femoral neck, hip, lumbar spine or whole body for adults aged >50 years, indicating osteopenia or osteoporosis; or for adults ≤50 years, a Z-score <−2.0 at these sites, indicating bone density below the expected range for age (9). Fat mass risk was classified as a body fat percentage of ≥35% for women or ≥25% for men, determined by body fat percentage derived by DXA (26). Lean mass risk was defined as reduced muscle mass and/or function, indicated by a low appendicular lean mass index (ALMI: women <5.5, men <7.0 kg/m^2^) and/or absolute maximum grip strength <25th percentile of established age-and sex-specific reference criteria (27, 28). Combined risk phenotypes were identified to capture overlapping risks, and included bone-fat risk, fat-lean risk and bone-lean risk. The most complex phenotype, bone-fat-lean risk, encompassed individuals with all three risks identified. Clinical terms such as osteoporosis or sarcopenia were not used to label phenotypes, as the definitions applied in this study were broader than clinical diagnostic criteria and were designed for a predominantly middle-aged population. In this age group, many individuals may present with early indicators of bone, fat or muscle risk that do not meet strict diagnostic thresholds established for older adults.

#### 2.3.3 Metabolic indicators

The most recent criteria, used to define metabolic syndrome (MetS) in this study, have been established by the Joint Interim Statement (JIS) in 2009 (29). JIS requires the presence of 3/5 risk factors: (i) population-specific abdominal obesity (waist circumference ≥80 cm for women and ≥94 cm for men in sub-Saharan African populations); (ii) fasting triglycerides ≥1.7 mmol/L; (iii) reduced high-density lipoprotein cholesterol (HDL-C; <1.3 mmol/L in women and <1.0 mmol/L in men); (iv) raised blood pressure (systolic ≥130 mmHg and/or diastolic ≥85 mmHg, or on treatment for hypertension); and (v) elevated fasting glucose (≥5.6 mmol/L, equivalent to glycated haemoglobin (HbA1c) ≥ 5.7% or on treatment for hyperglycaemia) (29, 30).

Waist circumference was measured to determine abdominal obesity, to the nearest 0.1 cm using a non-extensible steel tape (Seca 201, Surgical SA; Johannesburg, South Africa) according to standard protocols (31). Blood pressure was measured with a validated oscillometric monitor (Omron M6 AC; Hoofddorp, Netherlands), following a 5-min rest; three readings were taken with a 1-min interval in between, with the mean of the final 2 measures used for classification (32). Overnight fasting capillary samples were collected to determine blood lipids (triglycerides, HDL-C) and HbA1c using the point-of-care Alere Afinion AS100 analyser (Abbott Technologies; Abbott Park; IL, USA), following established procedures (33–35). Participants with elevated metabolic markers were referred for further medical screening. All teachers had access to employer-subsidised health insurance for follow-up care.

#### 2.3.4 Behavioural indicators

##### 2.3.4.1 Substance use

Substance use data were self-reported and included (i) current tobacco use or having quit within the previous 6 months and (ii) current alcohol consumption. As no safe threshold for alcohol consumption can be established for health, quantity and frequency of alcohol intake were not specified (36).

##### 2.3.4.2 Device-based physical activity

Physical activity was measured with tri-axial ActiGraph wGT3X-BT accelerometers (firmware 1.9.2; ActiGraph, Pensacola, FL, USA), initialised in ActiLife v6.13.4 at 30 Hz, idle-sleep enabled, local time GMT + 2. Monitors were worn on the hip for 7 consecutive days (5 weekdays, 2 weekend days), removed only for sleep and water-based activities. Raw acceleration was processed in R with GGIR v3.0.0 (intermediate day-level intensity summaries) using auto-calibration and the ENMO metric (Euclidean Norm Minus One; negative values set to zero; expressed in milligravity; m*g*) (37). Data were summarised in 60-s epochs and non-wear was identified by GGIR’s raw-signal algorithm using a 60-min evaluation window and 15-min resolution. Participants were included for weekly estimates if they had ≥3 valid wear days and ≥360 min/day average wear and daily summaries used the midnight-to-midnight window. Moderate-to-vigorous intensity physical activity (MVPA) was defined as ENMO ≥70 m*g* (38). Outcomes were MVPA time (min/week), and meeting the WHO guideline was defined as ≥150 min/week of MVPA (39).

##### 2.3.4.3 Grip strength

Grip strength was assessed using a hydraulic hand dynamometer (Saaehan; Tisselt, Belgium). Participants were seated in an upright position with their elbow at a 90° angle. Three trials were performed with each hand, alternating sides, with 30s rests between trials with the same hand. The maximum grip strength (kg) achieved across all trials, regardless of hand dominance, was used for analysis. Each participant’s age was matched to the closest category from published reference percentiles (27). Grip strength was classified as low (<25th percentile), average (25^th^–75th percentile), good (75^th^–90th percentile), or excellent (>90^th^ percentile) for age and sex (27).

##### 2.3.4.4 Weight-bearing exercise

Participants were asked to self-report current participation in weight-bearing exercise by responding yes or no to the question: “Are you currently participating in regular weight-bearing exercise?”

#### 2.3.5 Medical history

Medical history was obtained via self-report, capturing prior secondary osteoporosis diagnosis, osteoporosis treatment and treatment for cardiometabolic risk factors, including medication use for dyslipidaemia, hypertension and hyperglycaemia.

### 2.4 Ethics approval

The *KaziBantu* project adhered to the principles outlined in the Declaration of Helsinki, including its subsequent amendments. Ethics clearance for the study was granted by: (i) Nelson Mandela University Human Research Ethics Committee on 26 March 2018 (H19-HEA-HMS-001); (ii) Eastern Cape Department of Health on 5 June 2018 (EC_201804_007); (iii) Eastern Cape Department of Education on 9 May 2018 (no reference number provided); and (iv) Ethics Committee Northwest and Central Switzerland (EKNZ) on 1 March 2018 (R-2018-00047). The study was also registered with the International Standard Randomised Controlled Trial Number (ISRCTN) Registry (ISRCTN18485542). Prior to data collection, all participants provided written informed consent after receiving an explanation of the study’s objectives, potential risks and benefits.

### 2.5 Statistical analysis

For descriptive statistics, sample size (n), median (Med), interquartile range (IQR), mean (M), standard deviation (SD) and minimum and maximum values are reported for continuous variables. For categorical variables, frequencies (n) and percentages (%) are reported. Individual and combined phenotype risks were described in terms of frequency (n) and percentages (%). For prespecified primary prevalence estimates (bone-, fat-, lean mass risk, combined risk phenotypes, metabolic syndrome), we report 95% confidence intervals (95%CI) to indicate precision. Lastly, participant characteristics, including demographics, anthropometric, metabolic health, behavioural indicators and medical history are described by risk phenotype using summary statistics. All analyses were performed with R version 4.5.0 (2025-04-11; R Core Team; Vienna, Austria).

#### 2.5.1 Missing data imputation

As waist circumference data was required for MetS diagnosis, missing values were imputed using multiple imputation by chained equations (MICE) using the ‘mice’ package in R (v3.16.0). Predictive mean matching (PMM) was applied with height, weight, BMI, age and sex included as predictors (20 imputations, 200 iterations). Convergence was assessed using the Gelman-Rubin statistic ( $\hat{R}$ ). Descriptive statistics and correlation matrices of the original and imputed datasets were compared to ensure validity and consistency. A single imputed dataset was used for all subsequent analyses and reporting.

## 3 Results

### 3.1 Characteristics of participants

A total of 136 teachers were included in the analysis. The majority were women (85%, n = 116), reflecting the typical sex distribution among primary school teaching staff in South Africa. Teachers’ age ranged from 21–72 years (M = 47 years; SD = 11 years). Data availability varied across measures: all 136 participants completed the DXA scan, MetS could be classified for 132, and grip strength for 134 participants. Valid accelerometry meeting the wear-time criteria was available for 77 participants, with non-valid data arising primarily from insufficient wear days or daily wear time rather than device failure. A summary of descriptive statistics for key continuous and categorical variables is presented in Tables 2A–D. Full descriptive statistics for all continuous and categorical variables are provided in Supplementary Tables S1−S4.

**Table 2A:** Participant profile: summary of continuous indicators among public school teachers in mid-2019 in Gqeberha, South Africa.

Continuous variables	Sample size^i^	Median (Interquartile range)	Mean (Standard deviation)	Minimum–maximum
Demographic indicators				
Age (years in 2019)	136	50 (44–55)	47 (11)	21–72
Bone mass indicators				
Femoral neck bone mineral density (BMD) (g/cm^2^)	136	0.85 (0.77–0.94)	0.87 (0.13)	0.62–1.33
Femoral neck T-score	69	−0.2 (−0.9–0.6)	−0.1 (1.0)	−1.9–3.1
Femoral neck Z-score	136	0.6 (−0.3–1.5)	0.6 (1.2)	−1.9–4.4
Hip bone mineral density (BMD) (g/cm^2^)	136	1.00 (0.90–1.10)	1.00 (0.14)	0.73–1.45
Hip T-score	69	0.4 (−0.4–1.1)	0.4 (1.1)	−1.7–4.2
Hip Z-score	136	0.7 (−0.2–1.5)	0.7 (1.2)	−1.6–3.7
Lumbar spine bone mineral density (BMD) (g/cm^2^)	136	1.01 (0.89–1.09)	1.01 (0.14)	0.75–1.43
Lumbar spine T-score	69	−0.8 (−1.7–0.2)	−0.6 (1.2)	−2.7–2.3
Lumbar spine Z-score	136	0.1 (−0.9–1.0)	0.1 (1.3)	−2.7–3.8
Whole body bone mineral density (BMD) (g/cm^2^)	136	1.10 (1.05–1.17)	1.11 (0.10)	0.91–1.47
Whole body T-score	69	−0.4 (−1.2–0.7)	−0.3 (1.2)	−2.4–2.5
Whole body Z-score	136	−0.1 (−0.8–0.6)	−0.1 (1.0)	−2.5–2.4
Fat mass indicators				
Body fat (%)	136	44.6 (39.0–48.9)	42.5 (8.5)	13.4–54.4
Fat mass index (FMI) (kg/m^2^)	135	14.0 (10.1–17.9)	14.1 (5.3)	2.5–30.3
Lean mass indicators				
Appendicular lean mass index (ALMI) (kg/m^2^)	136	7.6 (6.4–8.5)	8.2 (4.2)	4.7–35.7
Anthropometric indicators				
Height (cm)	135	161.3 (155.3–165.0)	160.7 (7.3)	140.1–190.4
Weight^ii^ (kg)	136	84.8 (72.1–96.3)	85.7 (21.2)	47.3–160.5
Physical activity indicators				
Average ENMO (m*g*) ^iii^	77	13.27 (10.09–16.01)	13.4 (4.68)	4.79–27.6
MVPA^iv^ (≥70m*g*^v^; min/week)	77	119.00 (67.50–198.25)	146.0 (107.0)	1–611
Absolute maximum grip strength (kg)^vi^	134	34 (30–39)	34.7 (7.1)	22–66

**Table 2B:** Participant profile: summary of demographic, metabolic and behavioural categorical indicators, among public school teachers in mid-2019 in Gqeberha, South Africa.

Categorical variables	Category		Frequency^i^	Percentage^vii^
Demographic indicators				
Biological sex			136	100%
	Women		116	85.3%
	Men		20	14.7%
Race			136	100%
	Black		63	46.3%
	Coloured/Indian		66	48.5%
	White		7	5.2%
Metabolic indicators				
Metabolic syndrome^viii^			132	100%
	No		62	47%
	Yes		70	53%
Substance use indicators				
Tobacco use: “Do you currently smoke tobacco products, or have you quit smoking in the past 6 months?”			136	100%
	No		113	83.1%
	Yes		23	16.9%
Alcohol consumption: “Do you currently consume alcohol?”			136	100%
	No		88	64.7%
	Yes		48	35.3%
Physical activity indicators				
Met WHO physical activity guidelines^ix^			77	100%
	No		46	59.7%
	Yes		31	40.3%
Absolute grip strength classification			134	100%
	Low	< 25th percentile	18	13.4%
	Average	25^th^ - < 75th percentile	72	53.7%
	Good	75^th^ - < 90th percentile	19	14.2%
	Excellent	≥ 90^th^ percentile	25	18.7%
“Are you currently participating in regular weight-bearing exercise?”			102	100%
	No		65	63.7%
	Yes		37	36.3%

**Table 2C:** Participant profile: summary of body composition categorical indicators, among public school teachers in mid-2019 in Gqeberha, South Africa.

Categorical variables	Category				Frequency^i^	Percentage^vii^
Bone mass indicators					
Femoral neck T-score classification			69	100%
	Normal	≥ − 1.0	55	79.7%
	Osteopenia	−2.5 – −1.0	14	20.3%
	Osteoporosis	≤ − 2.5	0	0%
Femoral neck Z-score classification			136	100%
	Expected range for age	≥ − 2.0	136	100%
	Below expected range	< −2.0	0	0%
Hip T-score classification			69	100%
	Normal	≥ − 1.0	64	92.8%
	Osteopenia	−2.5 – −1.0	5	7.2%
	Osteoporosis	≤ − 2.5	0	0%
Hip Z-score classification			136	100%
	Expected range for age	≥ − 2.0	136	100%
	Below expected range	< −2.0	0	0%
Lumbar spine T-score classification			69	100%
	Normal	≥ − 1.0	38	55.1%
	Osteopenia	−2.5 – −1.0	29	42%
	Osteoporosis	≤ − 2.5	2	2.9%
Lumbar spine Z-score classification			136	100%
	Expected range for age	≥ − 2.0	129	94.9%
	Below expected range	< −2.0	7	5.1%
Whole body T-score classification			69	100%
	Normal	≥ − 1.0	47	68.1%
	Osteopenia	−2.5 – −1.0	22	31.9%
	Osteoporosis	≤ − 2.5	0	0%
Whole body Z-score classification			136	100%
	Expected range for age	≥ − 2.0	132	97.1%
	Below expected range	< −2.0	4	2.9%
Fat mass indicators					
Body fat (%) classification		Women	Men	136	100%
	Essential	10.0–13.9%	2.0–5.9%	0	0%
	Athletic	14.0–20.9%	6.0–13.9%	1	0.7%
	Fitness	21.0–24.9%	14.0–17.9%	1	0.7%
	Average	25.0–34.9%	18.0–24.9%	9	6.6%
	Obese	≥ 35.0%	≥ 25.0%	125	91.9%
Fat mass index (FMI) classification		Women	Men	135	100%
	Fat deficit	< 5	< 3	2	1.5%
	Normal	5–9	3–6	12	8.9%
	Excess fat	> 9–13	> 6–9	40	29.6%
	Obese class I	> 13–17	> 9–12	36	26.7%
	Obese class II	> 17-21	> 12–15	30	22.2%
	Obese class III	> 21	> 15	15	11.1%
Lean mass indicators					
Appendicular lean mass index (ALMI) (kg/m^2^) classification		Women	Men	136	100%
	Low	< 5.5	< 7.0	7	5.1%
	Normal	≥ 5.5	≥ 7.0	129	94.9%

**Table 2D:** Participant profile: summary of risk phenotype categorical indicators, among public school teachers in mid-2019 in Gqeberha, South Africa.

Categorical variables	Category	Frequency^i^	Percentage^vii^
Risk phenotype			
Bone mass risk: Adults > 50 years: T-score < −1.0 (osteopenia/osteoporosis in femoral neck, hip, lumbar spine, and/or whole body) Adults ≤ 50 years: Z-score < −2.0 (below expected range for age in femoral neck, hip, lumbar spine, and/or whole body)		134	100%
	No	94	70.1%
	Yes	40	29.9%
Fat mass risk: Dual-energy x-ray absorptiometry (DXA) scan derived body fat ≥ 35% for women and ≥ 25% for men		134	100%
	No	11	8.2%
	Yes	123	91.8%
Lean mass risk: Low appendicular lean mass index (ALMI) (women < 5.5 kg/m^2^; men < 7.0 kg/m^2^) and/or absolute maximum grip strength < 25^th^ percentile		134	100%
	No	113	84.3%
	Yes	21	15.7%
Bone-fat risk		134	100%
	No	97	72.4%
	Yes	37	27.6%
Fat-lean risk		134	100%
	No	116	86.6%
	Yes	18	13.4%
Bone-lean risk		134	100%
	No	125	93.3%
	Yes	9	6.7%
Bone-fat-lean risk		134	100%
	No	126	94%
	Yes	8	6%
Risk phenotype component count		134	100%
	0	6	4.5%
	1	80	59.7%
	2	40	29.9%
	3	8	6%

^i^Number of participants varies across variables due to differing amounts of missing data. ^ii^Measured with bioelectrical impedance scale (Tanita MC-580). ^iii^ENMO: Euclidean norm minus one, negative values set to zero (average acceleration metric from raw accelerometry), reported in milligravity units (mg). ^iv^ MVPA: moderate-to-vigorous intensity physical activity, defined as ENMO ≥70 mg using 60-s epochs. ^v^ mg: milligravity. ^vi^ Maximum value from 3 trials performed with each hand, regardless of hand dominance. ^vii^ Percentages (%) are calculated based on non-missing data for each variable. ^viii^ Metabolic syndrome if 3/5 criteria present (central obesity [imputed waist circumference values included], high triglycerides, low high density lipoprotein cholesterol, hypertension, and/or hyperglycaemia), defined by the Joint Interim Statement (JIS). ^ix^ Met World Health Organization (WHO) physical activity guideline of ≥150 min of MVPA per week.

### 3.2 Bone mass, fat mass, lean mass, and anthropometric profiles of teachers

#### 3.2.1 Characterisation of bone mass

BMD was largely within the normal range across sites (Tables 2A, C). At the femoral neck, median BMD was 0.85 g/cm^2^ (IQR 0.77–0.94). Among participants with T-scores available (>50 years; *n* = 69), 79.7% were normal and 20.3% osteopenic; none were osteoporotic. At the total hip, median BMD was 1.00 g/cm^2^ (IQR 0.90–1.10) with 92.8% normal and 7.2% osteopenic; none were osteoporotic. Lumbar spine BMD was similar (median 1.00 g/cm^2^; IQR 0.90–1.10), but T-scores were lower: 55.1% normal, 42.0% osteopenic and 2.9% osteoporotic. Z-scores were age-appropriate at most sites: all hip and femoral neck values were ≥ −2.0, whereas 5.1% at the spine fell below −2.0. Whole-body BMD median was 1.10 g/cm^2^ (IQR 1.05–1.17); 68.1% were normal and 31.9% osteopenic; none were osteoporotic. Median bone mass accounted for 2.7% of body mass (IQR 2.4–3.0) indicating that bone comprised a relatively small proportion of total body mass in this population (Supplementary Table S1).

#### 3.2.2 Characterisation of fat mass

The fat mass profile indicated substantial adiposity among teachers, underscoring potential health risks associated with obesity and its related conditions, alongside notable individual variability (Tables 2A, C; Supplementary Tables S1, S3). Median total fat mass was 35.1 kg (IQR 26.6–45.2; range 7.3–83.1) and median body fat percentage 44.6% (IQR 39.0–48.9); 91.9% (*n* = 125/136) met DXA body fat percentage obesity thresholds. The android/gynoid fat ratio median was 1.0 (IQR 0.9–1.1) with 90.4% (*n* = 123) exceeding sex-specific high-risk cut-offs. Fat mass index (FMI) median was 14.0 (IQR 10.1–17.9) with 29.6% in the excess-fat category and a further 60.0% in the obese category.

#### 3.2.3 Characterisation of lean mass

Lean mass indices were relatively symmetric (Tables 2A,C; Supplementary Tables S1, S3). The median total lean mass was 43.3 kg (IQR 38.5–50.4; range 28.1–98.3 kg). The median total lean mass index (TLMI) was 17.4 (14.8–19.2); classified, 45.6% were high, 39.7% normal, and 14.7% low, suggesting possible concerns related to insufficient lean body mass. The median ALMI was 7.6 kg/m^2^ (IQR 6.4–8.5); 94.9% were normal and 5.1% low, indicating a small pre-sarcopenia risk (28).

#### 3.2.4 Characterisation of anthropometric indicators

Anthropometric indicators are summarised in Table 2 and Supplementary Tables S1, S2. Median height was 161.3 cm (IQR 155.3–165.0) and median weight, measured with bioelectrical impedance, was 84.8 kg (IQR 72.1–96.3; range: 47.3–160.5 kg). Median BMI was 32.4 kg/m^2^ (IQR 27.1–38.6), indicating predominance of overweight/obesity with 1.5% classified as underweight, 11.9% normal, 23.7% overweight, and 63% obese class I-III. The median fat-to-muscle ratio (FMR) was 0.84 (IQR 0.67–1.00); 91.9% exceeded sex-specific high-risk cut-offs. These findings underscore the high prevalence of excess adiposity in this teacher population.

### 3.3 Prevalence and clustering of risk phenotypes

Adverse body composition phenotypes were common (Tables 2A, D; Supplementary Tables S1, S3). Fat mass risk was present in 91.8% (*n* = 123/134; 95%CI 85.8–95.8%) using DXA body fat percentage cut-offs (women ≥35%; men ≥25%). Lean mass risk affected 15.7% (*n* = 21; 95%CI 10–23%) based on low ALMI and/or reduced grip strength. Bone mass risk (osteopenia/osteoporosis if >50 years, or Z-score <−2.0 if ≤50 years) was identified in 29.9% (*n* = 40; 95%CI 22.3–38.4%) at one or more sites.

For combined risk phenotypes, participants could contribute to multiple combinations to reflect overlap. Fat-lean risk occurred in 13.4% (*n* = 18; 95%CI 8.2–20.4%), bone-fat risk in 27.6% (*n* = 37; 95%CI 20.2–36%), and bone-lean risk in 6.7% (*n* = 9; 95%CI 3.1–12.4%); notably 6% (*n* = 8; 95%CI 2.6–11.4%) had the bone-fat-lean phenotype. Only 4.5% (*n* = 6) had none of the body composition risk phenotypes, indicating a clustering of body composition-related health risks among teachers in this setting, with likely substantial impact on their physical function, metabolic health and long-term well-being.

Figure 1 (UpSet plot) visualises the prevalence and intersections of fat-, lean-, and bone-mass risk, showing mutually exclusive phenotypes where each participant is assigned to a single group based on their unique risk combination. This view enables between-phenotype comparisons that require exclusive membership. We present both representations because they address complementary questions: combination prevalence captures overlap and co-occurrence, whereas mutually exclusive groups define discrete categories for subsequent analyses.

**Figure 1:**
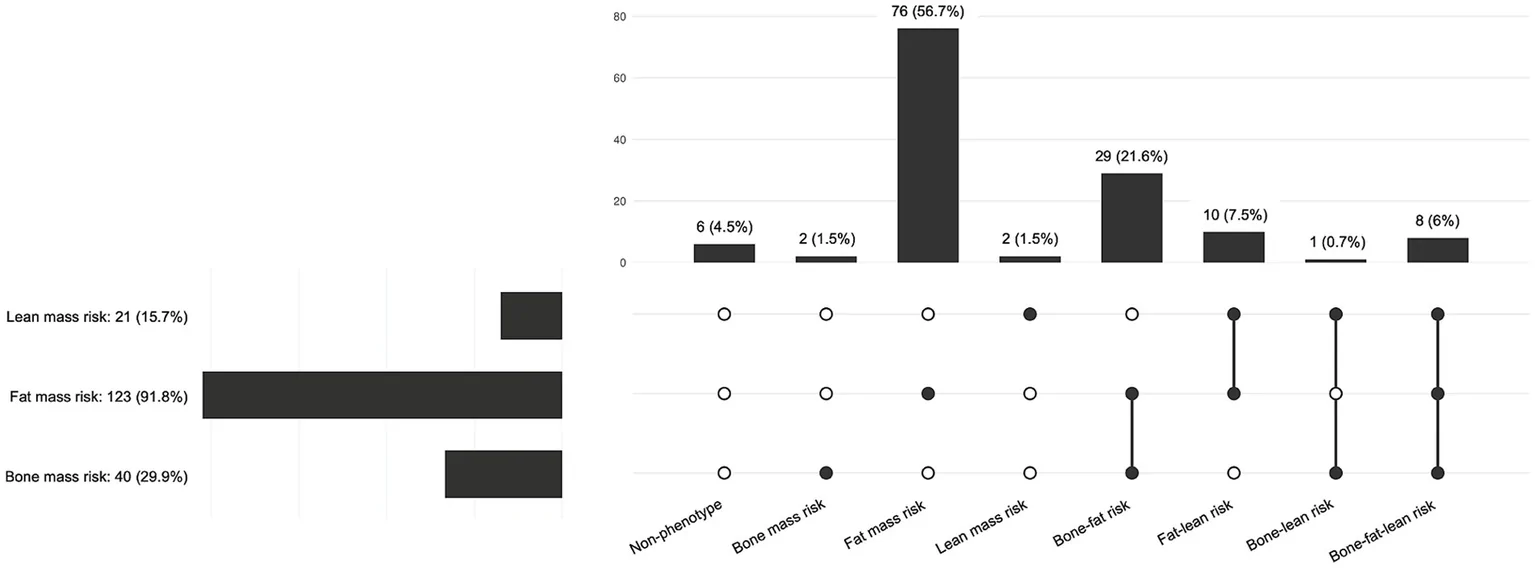
UpSet analysis of the prevalence and intersection of bone, fat and lean mass risk among public school teachers in Gqeberha, South Africa (mid-2019).

### 3.4 Descriptive profile of demographic, anthropometric, metabolic and behavioural characteristics by risk phenotype

Of 136 participants assessed, 134 were classified into body composition phenotype groups. The most common phenotype was fat mass risk (*n* = 76, 56.7%), followed by bone-fat risk (*n* = 29, 21.6%), fat-lean risk (*n* = 10, 7.5%), bone-fat-lean risk (*n* = 8, 6.0%), and a small non-phenotype group (*n* = 6, 4.5%). Residual categories were uncommon (bone mass risk *n* = 2, lean mass risk *n* = 2, bone-lean risk *n* = 1). Descriptive comparisons focus on the five main groups to contextualise how phenotype status varies across demographic (age, sex, income, education), anthropometric and DXA-derived indicators, metabolic markers (including metabolic syndrome), substance use, and physical activity. Because availability differed by domain (notably device-based physical activity), denominators are reported within the supplementary tables, and interpretations for small subgroups are cautious. Summary statistics are provided in Supplementary Tables S5−S14.

#### 3.4.1 Bone-fat-lean risk

Participants in the most complex phenotype (*n* = 8) comprised predominantly middle-aged participants (median 54 years), largely Coloured or Indian (75%), with mainly middle household income (75%) and diploma-level education (87.5%). This group showed a consistent pattern of relatively high adiposity compared with muscle mass (100% elevated FMR ratio), and mean fat mass (30.9 kg) exceeding what would be expected relative to mean lean mass (42.5 kg). Cardiometabolic risk clustered strongly: MetS was present in 62.5%, the highest proportion of all risk phenotypes, with high prevalence of hypertension (75%), and elevated triglycerides (85.7%). Behavioural indicators suggested lower than desired protective behaviours: although no reported tobacco use, 37.5% consumed alcohol, and few reported regular weight-bearing exercise (28.6%). Physical activity indicators were consistently low where measured: device-based activity was only available for 3 participants, and none met WHO MVPA guidelines. Functional strength was also low: grip strength was predominantly classified <25th age and sex percentile (87.5%). No one reported secondary osteoporosis or osteoporosis treatment, while cardiometabolic medication use was limited (25% treated for hypertension; 25% for hyperglycaemia; none for dyslipidaemia), which could indicate under-diagnosis and/or undertreatment of cardiometabolic risk despite the high burden hypertriglyceridaemia in this phenotype.

#### 3.4.2 Fat-lean risk

The fat-lean risk phenotype comprised 10 participants (7.5%), mainly middle-aged (median 49 years), with 60% categorised as Coloured or Indian and 40% as Black; household income was mostly middle (70%), and education spanned diploma to postgraduate levels. As defined, all participants had elevated adiposity relative to muscle mass (median FMR 0.7), with BMI largely in the overweight/obesity range (80%) and mean body mass 79.3 kg (mean fat mass 31.6 kg; mean lean mass 45.6 kg). Cardiometabolic risk was common: metabolic syndrome was present in 50%, hypertension (50%), hyperglycaemia (50%), and elevated triglycerides (55.6%). Behavioural and activity indicators were mixed: 20% reported tobacco use and 50% alcohol use, while device-based physical activity (available for 8/10) suggested 62.5% met WHO MVPA guidelines (median 162.5 MVPA min/week), yet functional strength was low (90% low grip strength) and self-reported weight-bearing exercise was uncommon (20%). Cardiometabolic medication use was modest (20% treated for dyslipidaemia, hypertension, and hyperglycaemia).

#### 3.4.3 Fat mass risk

The largest risk phenotype group, fat mass, comprised 76 participants (56.7%) predominantly middle-aged women (median age 48 years; 89.5% women; 43.4% Black; 51.3% Coloured/Indian), and socioeconomic indicators suggesting relative constraint (46.1% lower income, and diploma-level education most common). By definition, adiposity was high in all participants (median FMR ratio 0.9), with median body mass 86.3 kg and fat mass 39 kg, and BMI concentrated in obesity (72.1%; only 4.0% normal BMI). Cardiometabolic risk was substantial: continuous markers were broadly elevated (e.g., median waist 99.7 cm; HbA1c 5.5%), over half met metabolic syndrome criteria (56.2%), and this risk phenotype also contained the majority of participants with 5/5 MetS classification criteria (6/9 in the total sample). Substance use was common (18.4% tobacco; 39.5% alcohol). Physical activity and function were heterogeneous: among those with valid accelerometry (*n* = 37), 40.5% met WHO MVPA guidelines (median 119 MVPA min/week), self-reported weight-bearing exercise was reported by 36.8%, and grip strength was generally preserved (none classified as low; most average or excellent). No one reported secondary osteoporosis or osteoporosis treatment, while cardiometabolic medication use was present but limited (10.5% dyslipidaemia, 15.8% hypertension, 5.3% hyperglycaemia treatment).

#### 3.4.4 Bone-fat risk

The second largest risk phenotype group, bone-fat, (*n* = 29; 21.6%) comprised almost exclusively middle-aged women (96.6%; median age 56 years), with most participants categorised as Black (72.4%) and reporting lower or middle household income (48.3 and 37.9%); diploma-level education was most common (65.5%). Body composition reflected concurrent adiposity and bone risk: mean body mass was 78.6 kg with mean fat mass 35.6 kg, and all exceeded the FMR risk cut-off (median 0.9), with most in the overweight/obesity range (62% obese). Cardiometabolic risk clustered substantially, with central obesity prevalent (86.2%), alongside hypertension (63%), hyperglycaemia (46.4%), and elevated triglycerides (44.8%), and over half meeting metabolic syndrome criteria (53.6%). Physical activity was low for many with available accelerometry (*n* = 21), with 33.3% meeting WHO MVPA guidelines (median 115 MVPA min/week); grip strength was generally preserved (no low classifications; most average), while self-reported weight-bearing exercise was uncommon (23.8%). Tobacco use (10.3%) and alcohol consumption (13.8%) were relatively infrequent, no one reported secondary osteoporosis or osteoporosis treatment, and treatment for dyslipidaemia, hypertension, and hyperglycaemia was reported by only 13.8% each, despite the observed metabolic burden.

#### 3.4.5 Non-phenotype

The non-phenotype group was small (*n* = 6) and as expected, younger than the risk phenotypes (median age 38 years), with a higher proportion of men than most other groups and a diverse racial composition (50.0% Coloured/Indian; 33.3% White; 16.7% Black). Participants largely reported middle-to-higher household income and high educational attainment (all university/postgraduate). Body composition was comparatively favourable, with lower body and fat mass and a normal FMR for all participants (median 0.4), alongside a BMI profile that was mostly normal weight (50.0%) with fewer obesity classifications. Cardiometabolic risk was generally lower but not absent: one-third met criteria for central obesity and hypertension, and metabolic syndrome was present in 33.3% (2/6), with component counts ranging from 1 to 3. Behavioural indicators were not consistently protective, with comparatively high tobacco (33.3%) and alcohol use (66.7%), and limited device-based MVPA data suggesting low typical activity (median MVPA 42.7 min/week; 1/3 meeting WHO guidelines). Although weight-bearing exercise and grip strength were relatively favourable, the behavioural profile may still represent an early risk pathway, potentially increasing the likelihood of progression towards higher risk phenotypes and cardiometabolic clustering over time. No one reported secondary osteoporosis or osteoporosis treatment, and cardiometabolic medication use was rare; interpretation should be cautious given the small sample and limited accelerometry coverage.

## 4 Discussion

The study characterised bone, adiposity, and lean mass in a cohort of teachers working in under-resourced public schools. To our knowledge, this is the first DXA-based body composition study from the Eastern Cape province and the first of South African school staff. In 134 apparently healthy teachers, we quantified individual and combined risk phenotypes and examined their distribution across cardiometabolic and sociodemographic indicators. Body composition risk was prevalent, and approximately 1 in 17 teachers had the highest risk phenotype: bone-fat-lean risk. Risk phenotypes clustered with demographic and metabolic markers, with adverse profiles coinciding with higher multimorbidity and poorer metabolic health. Our detailed stratification underscores the importance of recognising combined risk phenotypes for targeted prevention and intervention strategies.

DXA access in South Africa is limited as resources prioritise infectious, maternal and child health, and a growing NCD burden; consequently, DXA-based body composition data in South Africa remain scarce. The largest DXA study in the country to date (*n* = 2,264; tertiary hospital referrals in Gauteng province, 2006–2021) reported 33.1% osteopenia and 30.8% osteoporosis (40). In our community sample, 29.9% had osteopenia/osteoporosis or bone health below age-expected norms, lower than the hospital cohort but notable given the substantial contextual differences. As the first DXA-based study from the Eastern Cape, our findings should be interpreted in light of the province’s structural context. The province consistently ranks among South Africa’s poorest, with the highest official unemployment rate in recent releases, which intensifies deprivation and stress with downstream health consequences (41). In Nelson Mandela Bay, violent crime is extreme (the most dangerous city nationally and 9th globally) (42), and residential segregation is longstanding (the most segregated among South Africa’s largest metros) (43). Studying teachers in this high-stress, under-resourced setting, shaped by concentrated poverty, segregation, and crime, may therefore reveal body composition and cardiometabolic vulnerabilities that studies from better-resourced provinces obscure, and provides context-specific evidence to guide provincial policy and workplace health interventions for teachers.

Globally, osteoporosis prevalence is estimated at 18.3% (95%CI 16.2–20.7%), with minimal African representation (3/86 studies in a recent systematic review and meta-analysis) and none from South Africa (10). Evidence in teachers is limited, possibly because their occupation is not usually associated with bone health outcomes, however, a Pakistani study reported 46% osteoporosis in a teacher cohort, attributed to sedentary lifestyles and suboptimal micronutrient intake (44). In our cohort, 59.7% did not meet MVPA recommendations (vs 31.3% globally; 44.8% nationally), and only a third of participants reported regular weight-bearing exercise (45). This is concerning, given physical activity and weight-bearing exercise’s important role in promoting bone and muscle health and the fact that 15.7% of our sample were classified as also having lean mass risk. Targeted, culturally sensitive weight-bearing activity, especially for middle-aged women, could support bone and muscle health, and weight management. Poor bone health may, in part, reflect nutrition-related factors, reduced cutaneous vitamin D synthesis associated with darker skin pigmentation, and a higher prevalence of lactose intolerance among Black Africans, which may reduce calcium intake (46). Antiretroviral therapy (efavirenz and lopinavir/ritonavir combination) can further lower BMD, and HIV prevalence among public-school educators was 15.3% in 2016, suggesting potential relevance in this occupation (47). Taken together, these findings highlight a burden of low bone and lean mass in this occupational group and the need not only for broader screening, using low-cost tools such as quantitative ultrasound, clinical risk algorithms, or bioelectrical impedance, to identify at-risk individuals, but also for feasible, context-appropriate interventions in under-resourced school settings.

As one of the most common global health challenges, obesity was also strikingly prevalent in our sample: 91.9% of teachers (94.8% of women, 75.0% of men) met DXA-derived body fat percentage obesity thresholds, far exceeding recent South African estimates and projections for 2050, and consistent with the country’s leading obesity burden in Africa, particularly among women (11). Reflecting the Lancet Commission’s framing of obesity as a chronic disease of adiposity-related organ dysfunction rather than excess weight alone, 14.9% (*n* = 20) met a conservative definition of clinical obesity using available metabolic markers (6). Beyond metabolic dysfunction, obesity has multiple implications, of which fall risk is particularly relevant to our study. One-third of participants had class II/III obesity, where fall risk and injuries are elevated, largely due to reduced mobility and postural instability, potentially compounded by low bone mass (6). The causes of obesity are multifactorial and not fully understood: alongside genetic, environmental, psychological, nutritional, and metabolic influences, the African context introduces unique cultural factors that may warrant consideration. Prior South African work, especially among Black women, links higher weight with social capital, while thinness could be associated with illness (48). Similar work conducted with teachers, in the same region as our study, has further highlighted socio-cultural barriers faced by Black women in being active, especially older women, including a lack of social support where they were actively discouraged from exercise, perceptions that physical activity was “not in my culture,” and traditional gender roles that prioritised household chores over leisure-time activity; in some cases, exercise was even associated with undesirable weight loss (49). These cultural perceptions could, however, contribute to the underestimation of personal weight status and the normalisation of obesity, delaying health-seeking behaviours and lifestyle changes. Further research is needed to assess whether these perceptions persist today: disease-related stigma may have lessened, but contemporary socio-cultural barriers (time constraints, competing domestic and work roles, safety and access, and shifting body-image norms) may now be equally or less influential. Effective responses should pair culturally sensitive community health education with structural strategies that improve food environments, reduce socioeconomic constraints, and expand safe, accessible spaces for physical activity.

The cohort was dominated by overlapping phenotypes: bone-fat was most common (21.6%), followed by fat-lean (7.5%), and bone-fat-lean risk (6%). Apart from a large subgroup with fat mass only risk (56.7%), isolated risks were uncommon, and only a handful of participants had no risk phenotype (6/134; 4.5%). Importantly, non-clinical phenotypes, as applied here, may flag emerging vulnerability before diagnostic thresholds are met, supporting early screening and prevention. Although we did not use clinical definitions (e.g., sarcopenia, sarcopenic obesity, osteosarcopenic obesity/adiposity), our categories are comparable and provide useful points of comparison. Osteosarcopenic obesity global prevalence estimates vary widely (0.8–66.3%) with older women affected disproportionately, and inconsistent definitions hinder firm conclusions (50). Evidence from Africa is scarce (0/39 studies in a recent review), underscoring the novelty and relevance of our findings (50). Risks in African populations may be compounded by the double burden of under- and over-nutrition, high obesity rates among women, and structural inequalities, including socioeconomic deprivation, inequitable access to healthcare, and disparities in food and built environments, alongside chronic infections such as HIV. Multimodal, culturally tailored strategies, such as resistance and weight-bearing activity plus protein- and micronutrient-rich diets, are promising and even low-cost community exercise and vitamin supplementation may mitigate disability in under-resourced settings (51).

The co-occurrence of low bone and muscle with excess adiposity has been associated with hypertension, dyslipidaemia, insulin resistance, hyperglycaemia, and chronic inflammation, underscoring systemic consequences beyond musculoskeletal health (5). Mechanistically, fat infiltration into bone and muscle, adipocyte dysfunction, and chronic inflammation can drive bone loss and reduced strength, resulting in functional decline (52). Our findings are consistent with these systemic links: over half of the cohort met MetS criteria (53%; *n* = 70; 95% CI 44.2–61.8%), indicating a substantial and largely undiagnosed cardiometabolic risk burden among teachers working in under-resourced communities. These results should also be interpreted alongside our companion analysis from the same cohort, which examined MetS as the primary outcome and found that older age and high total fat intake independently predicted MetS, while higher physical activity and higher household income were protective (53). The present study extends that work by showing how this cardiometabolic risk co-occurs with DXA-derived adiposity, lean mass, and bone-health phenotypes.

Together, these findings suggest that occupational-health responses should move beyond general individual behaviour-change messaging and instead target the modifiable factors identified in this cohort: improving diet quality, increasing feasible opportunities for physical activity during the working day, and addressing the income-related structural gradient through more equitable resourcing across schools. Practical strategies could include workplace-based screening and referral pathways that convert existing Government Employees Medical Scheme (GEMS) coverage into timely diagnosis and treatment; school-level measures such as protected time and safe spaces for physical activity; and employer-supported, culturally responsive health and nutrition education, particularly for the predominantly middle-aged, women workforce. These measures should be embedded within the education sector rather than relying solely on individual teacher agency. Doing so is likely to be both more effective and more equitable given the structural constraints teachers in under-resourced schools face.

These prevalence estimates should be read in light of who was available for study. As an actively employed workforce, teachers may be subject to a healthy worker effect: individuals with the most severe disease may already have left the profession, in which case our figures could understate the true burden among those who entered teaching. Among teachers still in post, non-participants could also have differed from participants in health status or workload, biasing prevalence in either direction. A related selection issue affects device-based physical activity, where the capacity to sustain uninterrupted accelerometer wear across a full week is itself shaped by work and home circumstances; valid wear was therefore unlikely to be completely at random and may have selected a non-representative subgroup, so physical activity estimates apply to those with valid wear rather than the full cohort, with the direction of bias uncertain. Designing for higher measurement completeness, and routinely checking who is selected into valid wear, would improve the representativeness of device-based data in comparable under-resourced settings.

### 4.1 Strengths and limitations

This study has several limitations that should be considered. Non-clinical definitions were used to categorise bone mass risk (including participants ≤50 years) and lean mass risk (based on reduced muscle mass and/or low grip strength), and no additional functional tests were performed. Detailed medical history was not obtained, and menopausal status, contraceptive use, HIV/AIDS diagnosis, and ART, was not taken, all of which may affect bone health. Dietary intake of relevant macro- and micronutrients (e.g., protein, calcium, and vitamin D) was also not assessed. Biological sex was self-reported and the cohort reflected the typically woman-dominant distribution of South African primary-school teachers. Small numbers within sex strata in this exploratory study however prevented reliable sex-disaggregated analyses and we therefore report pooled results only, which limits sex-specific and gender inferences. Finally, the cross-sectional design limits causal inference, and the relatively small occupation and geographic-specific sample limits generalisability. The Eastern Cape’s structural profile, concentrated poverty, the highest provincial unemployment, extreme violent crime, and entrenched residential segregation (described above), may amplify the body composition and cardiometabolic vulnerabilities observed here, so estimates should not be assumed to transfer to teachers in better-resourced provinces.

Despite these limitations, the study has notable strengths. We used DXA, the reference standard for detailed body composition analysis, to assess bone, fat, and lean mass in a group of teachers from under-resourced schools in the Eastern Cape province, where such data are missing. The study revealed a high prevalence of body composition risk phenotypes and multimorbidity, particularly linked with MetS, revealing a substantial, previously under-recognised occupational health burden.

## 5 Conclusion

This DXA-based study of public school teachers in an under-resourced South African setting shows a high prevalence of fat, lean, and bone risk, frequent overlap of phenotypes, and more than half meeting MetS criteria. The burden of multimorbidity has clear implications for health and well-being. As one of the first studies of its kind in Africa, our findings argue for screening and early detection, culturally appropriate prevention, and targeted occupational health strategies within the education sector, alongside longitudinal research to guide interventions and policy. Protecting teachers’ health, particularly in low-income communities, is essential not only for their well-being but also for sustaining their capacity to support learners and broader community development.

## Data Availability

The dataset underlying the present analysis is not publicly available due to ethical and participant privacy considerations. Data may be made available by the corresponding author on reasonable request, subject to appropriate ethical and data-sharing approvals.

## Glossary

A/G ratio: Android/gynoid ratio
AIDS: Acquired immunodeficiency syndrome
ALMI: Appendicular lean mass index
ART: Antiretroviral therapy
BMC: Bone mineral content
BMD: Bone mineral density
BMI: Body mass index
CI: Confidence interval
CV: Coefficient of variation
DAP: Dose area product
DXA: Dual-energy x-ray absorptiometry
EKNZ: Ethics Committee Northwest and Central Switzerland
ENMO: Euclidean norm minus one, with negative values set to zero
EWGSOP2: European Working Group on Sarcopenia in Older People
FMI: Fat mass index
FMR: Fat-to-muscle ratio
*g*: Gravitational unit
GEMS: Government Employees Medical Scheme
HbA1c: Glycated haemoglobin
HDL-C: High-density lipoprotein cholesterol
HIV: Human immunodeficiency virus
HPCSA: Health Professions Council of South Africa
IQR: Interquartile range
ISRCTN: International Standard Randomised Controlled Trial Number
JIS: Joint Interim Statement
LMICs: Low- and middle-income countries
M: Mean
Med: Median
MetS: Metabolic syndrome
m*g*: Milligravity
MICE: Multiple imputation by chained equations
MVPA: Moderate-to-vigorous intensity physical activity
n: Sample size / frequency
NCDs: Non-communicable diseases
NHANES: National Health and Nutrition Examination Survey
PMM: Predictive mean matching
$\hat{R}$: Gelman-Rubin statistic
SAHPRA: South African Health Products Regulatory Authority
SAMRC: South African Medical Research Council
SD: Standard deviation
TLMI: Total lean mass index
UNESCO: United Nations Educational, Scientific and Cultural Organization
WHO: World Health Organization
WHO STEPS: World Health Organization STEPwise approach to noncommunicable disease risk factor surveillance
