# Supplementary material for: Bone health, obesity, and multimorbidity risk among teachers in under-resourced South African public primary schools: results from a cross-sectional DXA study
Source: Front Public Health. 2026 Oct 9;14:1794604. doi: 10.3389/fpubh.2026.1794604 (PMC13690804; doi:10.3389/fpubh.2026.1794604)
Supplement: Supplementary file 1 [file Data_Sheet_1.docx]

Supplementary Material

## 1. Supplementary materials: STROBE checklist

| **Section/topic** | **Item number** | **Checklist item** | **Manuscript location/explanation** |
| --- | --- | --- | --- |
| **Title and abstract** | 1a | Indicate the study’s design with a commonly used term in the title or the abstract | Title and abstract (methods) |
|  | 1b | Provide in the abstract an informative and balanced summary of what was done and what was found | Abstract (methods, results) |
| **Introduction** | | | |
| Background/rationale | 2 | Explain the scientific background and rationale for the investigation being reported | 1. Introduction,  paragraph 1 |
| Objectives | 3 | State specific objectives, including any prespecified hypotheses | 1. Introduction,  paragraph 4 |
| **Methods** | | | |
| Study design | 4 | Present key elements of study design early in the paper | 2.1. Study design |
| Setting | 5 | Describe the setting, locations, and relevant dates, including periods of recruitment, exposure, follow-up, and data collection | 2.2. Setting and participants |
| Participants | 6a | Cross-sectional study – Give the eligibility criteria, and the sources and methods of selection of participants | 2.2. Setting and participants |
|  | 6b | Cohort study – For matched studies, give matching criteria and number of exposed and unexposed  Case-control study – For matched studies, give matching criteria and the number of controls per case | N/A |
| Variables | 7 | Clearly define all outcomes, exposures, predictors, potential confounders, and effect modifiers. Give diagnostic criteria, if applicable. | 2.3. Assessments |
| Data sources/ measurement | 8 | For each variable of interest, give sources of data and details of methods of assessment (measurement). Describe comparability of assessment methods if there is more than one group | 2.3. Assessments |
| Bias | 9 | Describe any efforts to address potential sources of bias | 2.2. Setting and participants,  2.3. Assessments, 2.5. Statistical analysis |
| Study size | 10 | Explain how the study size was arrived at | 2.2. Setting and participants |
| Quantitative variables | 11 | Explain how quantitative variables were handled in the analyses. If applicable, describe which groupings were chosen and why | 2.5. Statistical analysis |
| Statistical methods | 12a | Describe all statistical methods, including those used to control for confounding | 2.5. Statistical analysis |
|  | 12b | Describe any methods used to examine subgroups and interactions | Participant characteristics (demographic, anthropometric, metabolic, behavioural, medical history) are summarised descriptively by risk phenotype; no formal subgroup comparisons or interaction tests were undertaken |
|  | 12c | Explain how missing data were addressed | 2.5.1. Missing data imputation |
|  | 12d | Cross-sectional study – If applicable, describe analytical methods taking account of sampling strategy | Our sampling was purposive at school level, and all eligible staff were invited; analyses were unweighted and intended to describe participating schools rather than produce population-representative estimates. |
|  | 12e | Describe any sensitivity analyses | No sensitivity analyses were undertaken, as this was a descriptive cross-sectional study with limited sample size and no modelling. |
| **Results** | | | |
| Participants | 13a | Report numbers of individuals at each stage of study – e.g. numbers potentially eligible, examined for eligibility, confirmed eligible, included in the study, completing follow-up, and analysed | 2.2. Setting and participants  3.1. Characteristics of participants |
|  | 13b | Give reasons for non-participation at each stage | Invited 206; consented 168; completed DXA 136. The present cross-sectional analysis was restricted to participants with DXA data; data availability varied across other measures. Reasons for non-participation were due to non-response/scheduling. |
|  | 13c | Consider use of a flow diagram | N/A |
| Descriptive data | 14a | Give characteristics of study participants (e.g. demographic, clinical, social) and information on exposures and potential confounders | 3.1. Characteristics of participants |
|  | 14b | Indicate number of participants with missing data for each variable of interest | 3.1. Characteristics of participants |
|  | 14c | Cohort study – Summarise follow-up time (e.g., average and total amount) | N/A |
| Outcome data | 15 | Cross-sectional study – Report numbers of outcome events or summary measures | 3.1. Characteristics of participants |
| Main results | 16a | Give unadjusted estimates and, if applicable, confounder-adjusted estimates and their precision (e.g., 95% confidence interval). Make clear which confounders were adjusted for and why they were included | Unadjusted estimates (prevalence of each risk/phenotype) are reported with 95%CI in section 3.3. Prevalence and clustering of risk phenotypes.  No confounder-adjusted estimates were produced, as this was a descriptive analysis with no prespecified regression modelling. |
|  | 16b | Report category boundaries when continuous variables were categorized | 3.1. Characteristics of participants |
|  | 16c | If relevant, consider translating estimates of relative risk into absolute risk for a meaningful time period | N/A |
| Other analyses | 17 | Report other analyses done – e.g. analyses of subgroups and interactions, and sensitivity analyses | N/A |
| **Discussion** | | | |
| Key results | 18 | Summarise key results with reference to study objectives | 4. Discussion,  paragraph 1 |
| Limitations | 19 | Discuss limitations of the study, taking into account sources of potential bias or imprecision. Discuss both direction and magnitude of any potential bias. | 4. Discussion,  4.1. Strengths and limitations |
| Interpretation | 20 | Give a cautious overall interpretation of results considering objectives, limitations, multiplicity of analyses, results from similar studies, and other relevant evidence | 4. Discussion,  paragraph 2-6 |
| Generalisability | 21 | Discuss the generalisability (external validity) of the study results | 4. Discussion,  4.1. Strengths and limitations |
| **Other information** | | | |
| Funding | 22 | Give the source of funding and the role of the funders for the present study and, if applicable, for the original study on which the present article is based | Funders paragraph |

## 2. Supplementary tables

**Supplement table S1: Participant profile: all continuous indicators among public school teachers in mid-2019 in Gqeberha, South Africa**

| **Continuous variables** | **Sample size^i^** | **Median**  **(Interquartile range)** | **Mean**  **(Standard deviation)** | **Minimum –**  **maximum** |
| --- | --- | --- | --- | --- |
| **Demographic indicators** |  |  |  |  |
| Age (years in 2019) | 136 | 50 (44-55) | 47 (11) | 21-72 |
| **Bone mass indicators** |  |  |  |  |
| Femoral neck area (cm^2^) | 136 | 4.78 (4.56-5.05) | 4.78 (0.49) | 2.86-5.95 |
| Femoral neck bone mineral content (BMC) (g) | 136 | 4.04 (3.61-4.54) | 4.14 (0.79) | 2.76-6.31 |
| Femoral neck bone mineral density (BMD) (g/cm^2^) | 136 | 0.85 (0.77-0.94) | 0.87 (0.13) | 0.62-1.33 |
| Femoral neck T-score | 69 | -0.2 (-0.9-0.6) | -0.1 (1.0) | -1.9-3.1 |
| Femoral neck Z-score | 136 | 0.6 (-0.3-1.5) | 0.6 (1.2) | -1.9-4.4 |
| Hip area (cm^2^) | 136 | 32.20 (29.66-35.24) | 33.26 (5.34) | 24.41-55.61 |
| Hip bone mineral content (BMC) (g) | 136 | 32.18 (27.38-36.25) | 33.39 (8.16) | 17.85-68.00 |
| Hip bone mineral density (BMD) (g/cm^2^) | 136 | 1.00 (0.90-1.10) | 1.00 (0.14) | 0.73-1.45 |
| Hip T-score | 69 | 0.4 (-0.4-1.1) | 0.4 (1.1) | -1.7-4.2 |
| Hip Z-score | 136 | 0.7 (-0.2-1.5) | 0.7 (1.2) | -1.6-3.7 |
| Lumbar spine area (cm^2^) | 136 | 57.14 (51.73-62.07) | 57.10 (7.46) | 30.06-82.17 |
| Lumbar spine bone mineral content (BMC) (g) | 136 | 58.73 (46.45-64.76) | 57.55 (12.26) | 31.82-94.73 |
| Lumbar spine bone mineral density (BMD) (g/cm^2^) | 136 | 1.01 (0.89-1.09) | 1.01 (0.14) | 0.75-1.43 |
| Lumbar spine T-score | 69 | -0.8 (-1.7-0.2) | -0.6 (1.2) | -2.7-2.3 |
| Lumbar spine Z-score | 136 | 0.1 (-0.9-1.0) | 0.1 (1.3) | -2.7-3.8 |
| Whole body area (cm^2^) | 136 | 1978.4 (1856.8-2105.7) | 1986.8 (176.6) | 1578.5-2505.6 |
| Whole body bone mineral content (BMC) (g) | 136 | 2156.3 (1973.5-2422.3) | 2217.8 (362.7) | 1508.3-3592.8 |
| Whole body bone mineral density (BMD) (g/cm^2^) | 136 | 1.10 (1.05-1.17) | 1.11 (0.10) | 0.91-1.47 |
| Whole body T-score | 69 | -0.4 (-1.2-0.7) | -0.3 (1.2) | -2.4-2.5 |
| Whole body Z-score | 136 | -0.1 (-0.8-0.6) | -0.1 (1.0) | -2.5-2.4 |
| Bone mass (%) | 136 | 2.7 (2.4-3.0) | 2.7 (0.5) | 1.9-4.3 |
| **Fat mass indicators** |  |  |  |  |
| Total fat mass (g) | 136 | 35120 (26642-45205) | 36482 (13810) | 7270-83090 |
| Body fat (%) | 136 | 44.6 (39.0-48.9) | 42.5 (8.5) | 13.4-54.4 |
| Android/gynoid ratio | 136 | 1.00 (0.93-1.09) | 1.01 (0.17) | 0.63-1.67 |
| Fat mass index (FMI) (kg/m^2^) | 135 | 14.0 (10.1-17.9) | 14.1 (5.3) | 2.5-30.3 |
| **Lean mass indicators** |  |  |  |  |
| Total lean mass (g) | 136 | 43335 (38488-50418) | 45155 (10059) | 28050-98330 |
| Total lean mass index (TLMI) (kg/m^2^) | 136 | 17.4 (14.8-19.2) | 17.4 (3.0) | 11.7-27.1 |
| Appendicular lean mass index (ALMI) (kg/m^2^) | 136 | 7.6 (6.4-8.5) | 8.2 (4.2) | 4.7-35.7 |
| **Anthropometric indicators** |  |  |  |  |
| Height (cm) | 135 | 161.3 (155.3-165.0) | 160.7 (7.3) | 140.1-190.4 |
| Weight^ii^ (kg) | 136 | 84.8 (72.1-96.3) | 85.7 (21.2) | 47.3-160.5 |
| Body mass index (BMI) (kg/m^2^) | 135 | 32.4 (27.1-38.6) | 33.0 (7.6) | 17.5-58.5 |
| Body mass^iii^ (g) | 136 | 82205 (70495-94498) | 83855 (20729) | 46340-159970 |
| Combined lean mass and bone mineral content (BMC) (g) | 136 | 45505 (40738-52728) | 47373 (10354) | 29570-101920 |
| Fat-to-muscle ratio (FMR) | 136 | 0.84 (0.67-1.00) | 0.81 (0.24) | 0.16-1.25 |
| **Metabolic indicators** |  |  |  |  |
| Waist circumference^iv^ (cm) | 136 | 97.8 (83.8-106.2) | 95.6 (16.1) | 63.5-143.0 |
| Triglycerides (mmol/L) | 132 | 1.58 (1.13-2.16) | 1.84 (1.07) | 0.53-6.98 |
| High density lipoprotein cholesterol (HDL-C) (mmol/L) | 131 | 1.40 (1.17-1.62) | 1.42 (0.42) | 0.73-4.00 |
| Systolic blood pressure (mmHg) | 132 | 124 (114-138) | 127 (19) | 93-182 |
| Diastolic blood pressure (mmHg) | 132 | 84 (76-92) | 84 (11) | 62-127 |
| Glycated haemoglobin (HbA1c) (%) | 134 | 5.5 (5.3-5.9) | 6.0 (1.5) | 4.7-13.1 |
| **Physical activity indicators** |  |  |  |  |
| Accelerometer wear valid days (n) | 77 | 5 (4-6) | 4.87 (1.03) | 3-6 |
| Average daily accelerometer wear time (min/day) | 77 | 810.64 (659.14-949.39) | 788.0 (186.0) | 385-1125 |
| Average ENMO (m*g*)^v^ | 77 | 13.27 (10.09-16.01) | 13.4 (4.68) | 4.79-27.6 |
| Average MVPA^vi^ time (min/week) | 77 | 119.00 (67.50-198.25) | 146.0 (107.0) | 1-611 |
| Absolute maximum grip strength (kg)^vii^ | 134 | 34 (30-39) | 34.7 (7.1) | 22-66 |

**Supplement table S2: Participant profile: all demographic and anthropometry categorical indicators, among public school teachers in mid-2019 in Gqeberha, South Africa**

| **Categorical variables** | **Category** | | | | | **Frequency^i^** | **Percentage^viii^** |
| --- | --- | --- | --- | --- | --- | --- | --- |
| **Demographic indicators** |  | | | | |  |  |
| Biological sex |  | | | | | 136 | 100% |
|  | Women | | | | | 116 | 85.3% |
|  | Men | | | | | 20 | 14.7% |
| Natural age categories |  | | | | | 136 | 100% |
|  | Young adult | | | 18-29 years | | 15 | 11.0% |
|  | Adult | | | 30-44 years | | 25 | 18.4% |
|  | Middle age | | | 45-64 years | | 95 | 69.9% |
|  | Senior | | | ≥ 65 years | | 1 | 0.7% |
| Race |  | | | | | 136 | 100% |
|  | Black | | | | | 63 | 46.3% |
|  | Coloured and Indian | | | | | 66 | 48.5% |
|  | White | | | | | 7 | 5.2% |
| Tertiary education attainment |  | | | | | 130 | 100% |
|  | Diploma | | | | | 70 | 53.9% |
|  | University degree | | | | | 44 | 33.9% |
|  | Postgraduate degree | | | | | 16 | 12.3% |
| Average household monthly income |  | | | | | 136 | 100% |
|  | Lower | ≤ R 20,000 | | | | 55 | 40.4% |
|  | Middle | R20,001-R40,000 | | | | 60 | 44.1% |
|  | Higher | > R40,000 | | | | 21 | 15.4% |
| **Anthropometric indicators** |  | | | | |  |  |
| Body mass index (BMI) (kg/m^2^) classification |  | | | | | 135 | 100% |
|  | Underweight | | <18.5 | | | 2 | 1.5% |
|  | Normal | | 18.5-24.9 | | | 16 | 11.9% |
|  | Overweight | | 25-29.9 | | | 32 | 23.7% |
|  | Obese class I | | 30-34.9 | | | 33 | 24.4% |
|  | Obese class II | | 35-39.9 | | | 27 | 20.0% |
|  | Obese class III | | ≥40 | | | 25 | 18.5% |
| Fat-to-muscle ratio (FMR) classification |  | | Women | | Men | 136 | 100% |
|  | Normal | | < 0.50 | | < 0.35 | 11 | 8.1% |
|  | Elevated risk | | ≥ 0.50 | | ≥ 0.35 | 125 | 91.9% |

**Supplement table S3: Participant profile: all body composition categorical indicators, among public school teachers in mid-2019 in Gqeberha, South Africa**

| **Categorical variables** | **Category** | | | | **Frequency^i^** | **Percentage^ix^** |
| --- | --- | --- | --- | --- | --- | --- |
| **Bone mass indicators** |  | | | |  |  |
| Femoral neck T-score classification |  | | |  | 69 | 100% |
|  | Normal | | | ≥ -1.0 | 55 | 79.7% |
|  | Osteopenia | | | -2.5 – -1.0 | 14 | 20.3% |
|  | Osteoporosis | | | ≤ -2.5 | 0 | 0% |
| Femoral neck Z-score classification |  | | |  | 136 | 100% |
|  | Expected range for age | | | ≥ -2.0 | 136 | 100% |
|  | Below expected range | | | < -2.0 | 0 | 0% |
| Hip T-score classification |  | | |  | 69 | 100% |
|  | Normal | | | ≥ -1.0 | 64 | 92.8% |
|  | Osteopenia | | | -2.5 – -1.0 | 5 | 7.2% |
|  | Osteoporosis | | | ≤ -2.5 | 0 | 0% |
| Hip Z-score classification |  | | |  | 136 | 100% |
|  | Expected range for age | | | ≥ -2.0 | 136 | 100% |
|  | Below expected range | | | < -2.0 | 0 | 0% |
| Lumbar spine T-score classification |  | | |  | 69 | 100% |
|  | Normal | | | ≥ -1.0 | 38 | 55.1% |
|  | Osteopenia | | | -2.5 – -1.0 | 29 | 42% |
|  | Osteoporosis | | | ≤ -2.5 | 2 | 2.9% |
| Lumbar spine Z-score classification |  | | |  | 136 | 100% |
|  | Expected range for age | | | ≥ -2.0 | 129 | 94.9% |
|  | Below expected range | | | < -2.0 | 7 | 5.1% |
| Whole body T-score classification |  | | |  | 69 | 100% |
|  | Normal | | | ≥ -1.0 | 47 | 68.1% |
|  | Osteopenia | | | -2.5 – -1.0 | 22 | 31.9% |
|  | Osteoporosis | | | ≤ -2.5 | 0 | 0% |
| Whole body Z-score classification |  | | |  | 136 | 100% |
|  | Expected range for age | | | ≥ -2.0 | 132 | 97.1% |
|  | Below expected range | | | < -2.0 | 4 | 2.9% |
| **Fat mass indicators** |  | | | |  |  |
| Body fat (%) classification |  | Women | | Men | 136 | 100% |
|  | Essential | 10.0-13.9% | | 2.0-5.9% | 0 | 0% |
|  | Athletic | 14.0-20.9% | | 6.0-13.9% | 1 | 0.7% |
|  | Fitness | 21.0-24.9% | | 14.0-17.9% | 1 | 0.7% |
|  | Average | 25.0-34.9% | | 18.0-24.9% | 9 | 6.6% |
|  | Obese | ≥ 35.0% | | ≥ 25.0% | 125 | 91.9% |
| Android/gynoid ratio classification |  | Women | | Men | 136 | 100% |
|  | Low risk | ≤ 0.8 | | ≤ 1.0 | 13 | 9.6% |
|  | High risk | > 0.8 | | > 1.0 | 123 | 90.4% |
| Fat mass index (FMI) classification |  | Women | | Men | 135 | 100% |
|  | Fat deficit | < 5 | | < 3 | 2 | 1.5% |
|  | Normal | 5-9 | | 3-6 | 12 | 8.9% |
|  | Excess fat | > 9-13 | | > 6-9 | 40 | 29.6% |
|  | Obese class I | > 13-17 | | > 9-12 | 36 | 26.7% |
|  | Obese class II | > 17-21 | | > 12-15 | 30 | 22.2% |
|  | Obese class III | > 21 | | > 15 | 15 | 11.1% |
| **Lean mass indicators** |  | | | |  |  |
| Total lean mass index (TLMI) (kg/m²) classification |  | | Women | Men | 136 | 100% |
|  | Low | | < 14.0 | < 16.5 | 20 | 14.7% |
|  | Normal | | 14.0-17.5 | 16.5-19.5 | 54 | 39.7% |
|  | High | | > 17.5 | > 19.5 | 62 | 45.6% |
| Appendicular lean mass index (ALMI) (kg/m²) classification |  | | Women | Men | 136 | 100% |
|  | Low | | < 5.5 | < 7.0 | 7 | 5.1% |
|  | Normal | | ≥ 5.5 | ≥ 7.0 | 129 | 94.9% |
| **Risk phenotype** |  | | | |  |  |
| Bone mass risk:  Adults > 50 years: T-score < -1.0 (osteopenia/osteoporosis in femoral neck, hip, lumbar spine, and/or whole body)  Adults ≤ 50 years: Z-score < -2.0 (below expected range for age in femoral neck, hip, lumbar spine, and/or whole body) |  | | | | 134 | 100% |
|  | No | | | | 94 | 70.1% |
|  | Yes | | | | 40 | 29.9% |
| Fat mass risk:  Dual-energy x-ray absorptiometry (DXA) scan derived body fat ≥ 35% for women and ≥ 25% for men |  | | | | 134 | 100% |
|  | No | | | | 11 | 8.2% |
|  | Yes | | | | 123 | 91.8% |
| Lean mass risk:  Low appendicular lean mass index (ALMI) (women < 5.5 kg/m²; men < 7.0 kg/m²) and/or absolute maximum grip strength < 25^th^ percentile |  | | | | 134 | 100% |
|  | No | | | | 113 | 84.3% |
|  | Yes | | | | 21 | 15.7% |
| Bone-fat risk |  | | | | 134 | 100% |
|  | No | | | | 97 | 72.4% |
|  | Yes | | | | 37 | 27.6% |
| Fat-lean risk |  | | | | 134 | 100% |
|  | No | | | | 116 | 86.6% |
|  | Yes | | | | 18 | 13.4% |
| Bone-lean risk |  | | | | 134 | 100% |
|  | No | | | | 125 | 93.3% |
|  | Yes | | | | 9 | 6.7% |
| Bone-fat-lean risk |  | | | | 134 | 100% |
|  | No | | | | 126 | 94% |
|  | Yes | | | | 8 | 6% |
| Risk phenotype component count |  | | | | 134 | 100% |
|  | 0 | | | | 6 | 4.5% |
|  | 1 | | | | 80 | 59.7% |
|  | 2 | | | | 40 | 29.9% |
|  | 3 | | | | 8 | 6% |

**Supplement table S4: Participant profile: all metabolic, behavioural and medical categorical indicators among public school teachers in mid-2019 in Gqeberha, South Africa**

| **Categorical variables** | **Category** | | **Frequency^i^** | **Percentage^xi^** |
| --- | --- | --- | --- | --- |
| **Metabolic indicators** |  | |  |  |
| Metabolic syndrome^ix^ |  | | 132 | 100% |
|  | No | | 62 | 47% |
|  | Yes | | 70 | 53% |
| Metabolic syndrome criteria for waist circumference:^iv^  ≥80 cm women; ≥94 cm men, in Sub-Saharan African |  | | 136 | 100% |
|  | No | | 29 | 21.3% |
|  | Yes | | 107 | 78.7% |
| Metabolic syndrome criteria for triglycerides:  ≥1.7 mmol/L or on treatment for dyslipidaemia |  | | 132 | 100% |
|  | No | | 72 | 54.5% |
|  | Yes | | 60 | 45.5% |
| Metabolic syndrome criteria for high density lipoprotein cholesterol (HDL-C):  <1.3 mmol/L women; <1.0 mmol/L men, or on treatment for dyslipidaemia |  | | 131 | 100% |
|  | No | | 83 | 63.4% |
|  | Yes | | 48 | 36.6% |
| Metabolic syndrome criteria for blood pressure:  systolic ≥130 mmHg and/or diastolic ≥85 mmHg or on treatment for hypertension |  | | 132 | 100% |
|  | No | | 58 | 43.9% |
|  | Yes | | 74 | 56.1% |
| Metabolic syndrome criteria for glucose:  fasting glucose ≥5.6 mmol/L (equivalent to glycated haemoglobin (HbA1c) ≥5.7%) or on treatment for hyperglycaemia |  | | 135 | 100% |
|  | No | | 83 | 61.5% |
|  | Yes | | 52 | 38.5% |
| Metabolic syndrome component count |  | | 126 | 100% |
|  | 0 | | 11 | 8.7% |
|  | 1 | | 22 | 17.5% |
|  | 2 | | 27 | 21.4% |
|  | 3 | | 29 | 23.0% |
|  | 4 | | 28 | 22.2% |
|  | 5 | | 9 | 7.1% |
| **Substance use indicators** |  | |  |  |
| Tobacco use:  “Do you currently smoke tobacco products, or have you quit smoking in the past 6 months?” |  | | 136 | 100% |
|  | No | | 113 | 83.1% |
|  | Yes | | 23 | 16.9% |
| Alcohol consumption:  “Do you currently consume alcohol?” |  | | 136 | 100% |
|  | No | | 88 | 64.7% |
|  | Yes | | 48 | 35.3% |
| **Physical activity indicators** |  | |  |  |
| Met WHO physical activity guidelines^x^ |  | | 77 | 100% |
|  | No | | 46 | 59.7% |
|  | Yes | | 31 | 40.3% |
| Absolute maximum grip strength classification |  | | 134 | 100% |
|  | Low | < 25^th^ percentile | 18 | 13.4% |
|  | Average | 25^th^ - < 75^th^ percentile | 72 | 53.7% |
|  | Good | 75^th^ - < 90^th^ percentile | 19 | 14.2% |
|  | Excellent | ≥ 90^th^ percentile | 25 | 18.7% |
| “Are you currently participating in regular weight-bearing exercise?” |  | | 102 | 100% |
|  | No | | 65 | 63.7% |
|  | Yes | | 37 | 36.3% |
| **Medical history indicators** |  | |  |  |
| Secondary osteoporosis diagnosis |  | | 114 | 100% |
|  | No | | 114 | 100% |
|  | Yes | | 0 | 0% |
| Treatment for osteoporosis |  | | 114 | 100% |
|  | No | | 114 | 100% |
|  | Yes | | 0 | 0% |
| Treatment for dyslipidaemia |  | | 136 | 100% |
|  | No | | 122 | 89.7% |
|  | Yes | | 14 | 10.3% |
| Treatment for hypertension |  | | 136 | 100% |
|  | No | | 115 | 84.6% |
|  | Yes | | 21 | 15.4% |
| Treatment for hyperglycaemia |  | | 136 | 100% |
|  | No | | 122 | 89.7% |
|  | Yes | | 14 | 10.3% |

**Supplement table S1, S2, S3 and S4 notes:**

^i^ Number of participants varies across variables due to differing amounts of missing data.

^ii^ Measured with bioelectrical impedance scale (Tanita MC-580).

^iii^ Measured with dual-energy X-ray absorptiometry (DXA) scan (Discovery Hologic DXA QDR 4500A).

^iv^ Imputed waist circumference values included.

^v^ ENMO: Euclidean norm minus one (average acceleration metric from raw accelerometry), reported in milligravity units (m*g*).

^vi^ MVPA: moderate-to-vigorous physical activity.

^vii^ Maximum value from 3 trials performed with each hand, regardless of hand dominance.

^viii^ Percentages (%) are calculated based on non-missing data for each variable.

^ix^ Metabolic syndrome if 3/5 criteria present (central obesity^iv^, high triglycerides, low high density lipoprotein cholesterol, hypertension, and/or hyperglycaemia), defined by the Joint Interim Statement (JIS).

^x^ Met World Health Organization (WHO) physical activity guideline of ≥150 mins of MVPA^vi^ per week.

#### Supplement table S5: Distribution of continuous demographic indicators by phenotype risk group among public primary school teachers in mid-2019 in Gqeberha, South Africa

|  | | **Age**  **(years)** | |
| --- | --- | --- | --- |
| **Phenotype** | **n^i^ (%)** | **Med^ii^ (IQR^iii^)** | **M^iv^ (SD^v^)** |
| Bone-fat-lean risk | 8  (6.0%) | 54.0  (53.8–54.0) | 51.2  (7.8) |
| Fat-lean risk | 10  (7.5%) | 49.0  (34.8–50.8) | 44.3  (11.0) |
| Fat mass risk | 76 (56.7%) | 48.0  (40.8–54.0) | 45.6  (10.6) |
| Bone-fat risk | 29 (21.6%) | 56.0  (52.0–58.0) | 54.4  (5.5) |
| Non-phenotype | 6  (4.5%) | 38.0  (26.2–49.0) | 37.3  (12.7) |
| Bone mass risk | 2  (1.5%) | 55.0  (54.5–55.5) | 55.0  (1.4) |
| Bone-lean risk | 1  (0.7%) | 45.0  (45.0–45.0) | 45.0  (—) |
| Lean mass risk | 2  (1.5%) | 23.0  (22.0–24.0) | 23.0  (2.8) |
| **Total** | **134 (100.0%)** | **50.0**  **(43.2–55.0)** | **47.1**  **(10.7)** |

^i^ Number of participants

^ii^ Median

^iii^ Interquartile range

^iv^ Mean

^v^ Standard deviation

#### Supplement table S6: Distribution of categorical demographic indicators by phenotype risk group among public primary school teachers in mid-2019 in Gqeberha, South Africa

|  | **Age**  **(Natural categories)** | | | | | **Biological sex** | | | **Race** | | | | **Average household monthly income** | | | | **Education attainment** | | | |
| --- | --- | --- | --- | --- | --- | --- | --- | --- | --- | --- | --- | --- | --- | --- | --- | --- | --- | --- | --- | --- |
| **Phenotype** | **n^i^ (%)**  **Total** | **Young adult** | **Adult** | **Middle age** | **Senior** | **n (%)**  **Total** | **Woman** | **Man** | **n (%)**  **Total** | **Black** | **Coloured or Indian** | **White** | **n (%)**  **Total** | **Lower^ii^** | **Middle^iii^** | **Higher^iv^** | **n (%)**  **Total** | **Diploma** | **University degree** | **Postgraduate degree** |
| Bone-fat-lean risk | 8  (6.0%) | 0  (0.0%) | 1 (12.5%) | 7 (87.5%) | 0  (0.0%) | 8  (6.0%) | 5 (62.5%) | 3 (37.5%) | 8  (6.0%) | 2 (25.0%) | 6  (75.0%) | 0 (0.0%) | 8  (6.0%) | 0  (0.0%) | 6 (75.0%) | 2 (25.0%) | 8  (6.2%) | 7 (87.5%) | 1 (12.5%) | 0  (0.0%) |
| Fat-lean risk | 10 (7.5%) | 1 (10.0%) | 2 (20.0%) | 7 (70.0%) | 0  (0.0%) | 10 (7.5%) | 7 (70.0%) | 3 (30.0%) | 10 (7.5%) | 4 (40.0%) | 6  (60.0%) | 0 (0.0%) | 10 (7.5%) | 2 (20.0%) | 7 (70.0%) | 1 (10.0%) | 9  (7.0%) | 4 (44.4%) | 3 (33.3%) | 2 (22.2%) |
| Fat mass risk | 76 (56.7%) | 10 (13.2%) | 20 (26.3%) | 46 (60.5%) | 0  (0.0%) | 76 (56.7%) | 68 (89.5%) | 8 (10.5%) | 76 (56.7%) | 33 (43.4%) | 39 (51.3%) | 4 (5.3%) | 76 (56.7%) | 35 (46.1%) | 28 (36.8%) | 13 (17.1%) | 71 (55.5%) | 35 (49.3%) | 28 (39.4%) | 8 (11.3%) |
| Bone-fat risk | 29 (21.6%) | 0  (0.0%) | 1  (3.4%) | 28 (96.6%) | 0  (0.0%) | 29 (21.6%) | 28 (96.6%) | 1  (3.4%) | 29 (21.6%) | 21 (72.4%) | 8  (27.6%) | 0 (0.0%) | 29 (21.6%) | 14 (48.3%) | 11 (37.9%) | 4 (13.8%) | 29 (22.7%) | 19 (65.5%) | 5 (17.2%) | 5 (17.2%) |
| Non-phenotype | 6  (4.5%) | 2 (33.3%) | 1 (16.7%) | 3 (50.0%) | 0  (0.0%) | 6  (4.5%) | 4 (66.7%) | 2 (33.3%) | 6  (4.5%) | 1 (16.7%) | 3  (50.0%) | 2 (33.3%) | 6  (4.5%) | 1 (16.7%) | 4 (66.7%) | 1 (16.7%) | 6  (4.7%) | 0  (0.0%) | 5 (83.3%) | 1 (16.7%) |
| Bone mass risk | 2  (1.5%) | 0  (0.0%) | 0  (0.0%) | 2 (100.0%) | 0  (0.0%) | 2  (1.5%) | 1 (50.0%) | 1 (50.0%) | 2  (1.5%) | 0  (0.0%) | 2 (100.0%) | 0 (0.0%) | 2  (1.5%) | 0  (0.0%) | 2 (100.0%) | 0  (0.0%) | 2  (1.6%) | 2 (100.0%) | 0  (0.0%) | 0  (0.0%) |
| Bone-lean risk | 1  (0.7%) | 0  (0.0%) | 0  (0.0%) | 1 (100.0%) | 0  (0.0%) | 1  (0.7%) | 0  (0.0%) | 1 (100.0%) | 1  (0.7%) | 0  (0.0%) | 1 (100.0%) | 0 (0.0%) | 1  (0.7%) | 0  (0.0%) | 1 (100.0%) | 0  (0.0%) | 1  (0.8%) | 1 (100.0%) | 0  (0.0%) | 0  (0.0%) |
| Lean mass risk | 2  (1.5%) | 2 (100.0%) | 0  (0.0%) | 0  (0.0%) | 0  (0.0%) | 2  (1.5%) | 1 (50.0%) | 1 (50.0%) | 2  (1.5%) | 0  (0.0%) | 1  (50.0%) | 1 (50.0%) | 2  (1.5%) | 1 (50.0%) | 1 (50.0%) | 0  (0.0%) | 2  (1.6%) | 1 (50.0%) | 1 (50.0%) | 0  (0.0%) |
| **Total** | **134 (100.0%)** | **15 (11.2%)** | **25 (18.7%)** | **94 (70.1%)** | **0**  **(0.0%)** | **134 (100.0%)** | **114 (85.1%)** | **20 (14.9%)** | **134 (100.0%)** | **61 (45.5%)** | **66 (49.3%)** | **7 (5.2%)** | **134 (100.0%)** | **53 (39.6%)** | **60 (44.8%)** | **21 (15.7%)** | **128 (100.0%)** | **69 (53.9%)** | **43 (33.6%)** | **16 (12.5%)** |

^i^ Number of participants

^ii^ Lower: ≤R 20,000

^iii^ Middle: R 20,001–40,000

^iv^ Higher: >R 40,000

#### Supplement table S7: Distribution of continuous anthropometric indicators by phenotype risk group among public primary school teachers in mid-2019 in Gqeberha, South Africa

|  | **Height**  **(cm)** | | | **Body mass**  **(g)** | | | **Fat mass**  **(g)** | | | **Bone mass**  **(g)** | | | **Lean mass**  **(g)** | | | **Fat-to-muscle ratio** | | |
| --- | --- | --- | --- | --- | --- | --- | --- | --- | --- | --- | --- | --- | --- | --- | --- | --- | --- | --- |
| **Phenotype** | **n^i^ (%)** | **Med^ii^ (IQR^iii^)** | **M^iv^ (SD^v^)** | **n (%)** | **Med**  **(IQR)** | **M**  **(SD)** | **n (%)** | **Med (IQR)** | **M**  **(SD)** | **n (%)** | **Med**  **(IQR)** | **M**  **(SD)** | **n (%)** | **Med**  **(IQR)** | **M**  **(SD)** | **n (%)** | **Med (IQR)** | **M (SD)** |
| Bone-fat-lean risk | 8  (6.0%) | 158.4 (153.3–164.5) | 159.6 (8.3) | 8  (6.0%) | 76075.0 (60455.0–85465.0) | 75415.0 (20581.7) | 8  (6.0%) | 26405.0 (22292.5–39790.0) | 30952.5 (13452.8) | 8  (6.0%) | 1943.3 (1757.1–2167.1) | 1948.8 (272.8) | 8  (6.0%) | 41410.0 (31277.5–54095.0) | 42511.2 (11858.8) | 8  (6.0%) | 0.7  (0.5–1.0) | 0.8  (0.3) |
| Fat-lean risk | 10  (7.5%) | 160.5 (156.8–166.9) | 160.4 (10.3) | 10  (7.5%) | 80910.0 (56315.0–91797.5) | 79283.0 (25399.5) | 10  (7.5%) | 26375.0 (20052.5–39217.5) | 31572.0 (14103.3) | 10  (7.5%) | 2188.3 (1862.7–2335.8) | 2153.9 (366.1) | 10  (7.5%) | 50310.0 (33552.5–53972.5) | 45555.0 (12942.8) | 10  (7.5%) | 0.7  (0.6–0.7) | 0.7  (0.2) |
| Fat mass risk | 75 (56.4%) | 161.5 (156.8–165.2) | 161.2 (6.4) | 76 (56.7%) | 86280.0 (75132.5–100272.5) | 90056.1 (20181.4) | 76 (56.7%) | 38965.0 (31227.5–48232.5) | 40998.4 (13132.6) | 76 (56.7%) | 2316.5 (2044.7–2524.1) | 2334.4 (337.0) | 76 (56.7%) | 45705.0 (40745.0–51297.5) | 46722.9 (8903.0) | 76 (56.7%) | 0.9  (0.7–1.0) | 0.9  (0.2) |
| Bone-fat risk | 29 (21.8%) | 159.3 (152.6–163.2) | 158.1 (6.7) | 29 (21.6%) | 79150.0 (69160.0–87070.0) | 78646.9 (12933.1) | 29 (21.6%) | 34850.0 (27620.0–43010.0) | 35610.7 (8957.5) | 29 (21.6%) | 1963.0 (1901.9–2085.9) | 1963.3 (182.0) | 29 (21.6%) | 40550.0 (37770.0–43200.0) | 41075.2 (5709.5) | 29 (21.6%) | 0.9  (0.7–1.0) | 0.9  (0.2) |
| Non-phenotype | 6  (4.5%) | 164.8 (161.2–167.3) | 167.4 (12.0) | 6  (4.5%) | 68295.0 (56820.0–77362.5) | 75080.0 (26747.4) | 6  (4.5%) | 16230.0 (14922.5–22112.5) | 18163.3 (4648.8) | 6  (4.5%) | 2321.5 (2163.1–2892.2) | 2583.7 (612.4) | 6  (4.5%) | 48270.0 (39510.0–56145.0) | 54333.3 (23081.4) | 6  (4.5%) | 0.4  (0.3–0.5) | 0.4  (0.1) |
| Bone mass risk | 2  (1.5%) | 161.6 (158.1–165.1) | 161.6 (9.9) | 2  (1.5%) | 52110.0 (50315.0–53905.0) | 52110.0 (5077.0) | 2  (1.5%) | 13085.0 (11967.5–14202.5) | 13085.0 (3160.8) | 2  (1.5%) | 2023.0 (1929.0–2116.9) | 2023.0 (265.7) | 2  (1.5%) | 37000.0 (34180.0–39820.0) | 37000.0 (7976.2) | 2  (1.5%) | 0.4  (0.3–0.4) | 0.4  (0.2) |
| Bone-lean risk | 1  (0.8%) | 167.2 (167.2–167.2) | 167.2  (—) | 1  (0.7%) | 49380.0 (49380.0–49380.0) | 49380.0 (—) | 1  (0.7%) | 9290.0 (9290.0–9290.0) | 9290.0 (—) | 1  (0.7%) | 1863.0 (1863.0–1863.0) | 1863.0 (—) | 1  (0.7%) | 38220.0 (38220.0–38220.0) | 38220.0 (—) | 1  (0.7%) | 0.2  (0.2–0.2) | 0.2  (—) |
| Lean mass risk | 2  (1.5%) | 167.5 (166.5–168.5) | 167.5 (3.0) | 2  (1.5%) | 50895.0 (49122.5–52667.5) | 50895.0 (5013.4) | 2  (1.5%) | 10155.0 (8712.5–11597.5) | 10155.0 (4080.0) | 2  (1.5%) | 2126.1 (2077.8–2174.4) | 2126.1 (136.6) | 2  (1.5%) | 38610.0 (35445.0–41775.0) | 38610.0 (8952.0) | 2  (1.5%) | 0.3  (0.2–0.3) | 0.3  (0.2) |
| **Total** | **133 (100.0%)** | **161.4 (155.4–165.1)** | **160.8 (7.3)** | **134 (100.0%)** | **82000.0 (70125.0–94552.5)** | **83783.9 (20874.8)** | **134 (100.0%)** | **34900.0 (26607.5–45395.0)** | **36393.1 (13891.1)** | **134 (100.0%)** | **2156.3 (1973.0–2410.8)** | **2217.5 (364.7)** | **134 (100.0%)** | **43120.0 (38462.5–50512.5)** | **45173.1 (10133.1)** | **134 (100.0%)** | **0.8**  **(0.7–1.0)** | **0.8**  **(0.2)** |

^i^ Number of participants

^ii^ Median

^iii^ Interquartile range

^iv^ Mean

^v^ Standard deviation

#### Supplement table S8: Distribution of categorical anthropometric indicators by phenotype risk group among public primary school teachers in mid-2019 in Gqeberha, South Africa

|  | **Body mass index** | | | | | | | **Fat-to-muscle ratio** | | |
| --- | --- | --- | --- | --- | --- | --- | --- | --- | --- | --- |
| **Phenotype** | **n^i^ (%)** | **Underweight** | **Normal** | **Overweight** | **Obesity class I** | **Obesity class II** | **Obesity class III** | **n (%)** | **Normal** | **Elevated risk** |
| Bone-fat-lean risk | 8  (6.0%) | 0  (0.0%) | 2  (25.0%) | 2  (25.0%) | 2  (25.0%) | 1  (12.5%) | 1  (12.5%) | 8  (6.0%) | 0  (0.0%) | 8  (100.0%) |
| Fat-lean risk | 10 (7.5%) | 0  (0.0%) | 2  (20.0%) | 3  (30.0%) | 2  (20.0%) | 1  (10.0%) | 2  (20.0%) | 10  (7.5%) | 0  (0.0%) | 10  (100.0%) |
| Fat mass risk | 75 (56.4%) | 0  (0.0%) | 3  (4.0%) | 18  (24.0%) | 20  (26.7%) | 17  (22.7%) | 17  (22.7%) | 76 (56.7%) | 0  (0.0%) | 76  (100.0%) |
| Bone-fat risk | 29 (21.8%) | 0  (0.0%) | 3  (10.3%) | 8  (27.6%) | 8  (27.6%) | 5  (17.2%) | 5  (17.2%) | 29 (21.6%) | 0  (0.0%) | 29  (100.0%) |
| Non-phenotype | 6  (4.5%) | 0  (0.0%) | 3  (50.0%) | 1  (16.7%) | 1  (16.7%) | 1  (16.7%) | 0  (0.0%) | 6  (4.5%) | 6 (100.0%) | 0  (0.0%) |
| Bone mass risk | 2  (1.5%) | 0  (0.0%) | 2 (100.0%) | 0  (0.0%) | 0  (0.0%) | 0  (0.0%) | 0  (0.0%) | 2  (1.5%) | 2 (100.0%) | 0  (0.0%) |
| Bone-lean risk | 1  (0.8%) | 1  (100.0%) | 0  (0.0%) | 0  (0.0%) | 0  (0.0%) | 0  (0.0%) | 0  (0.0%) | 1  (0.7%) | 1 (100.0%) | 0  (0.0%) |
| Lean mass risk | 2  (1.5%) | 1  (50.0%) | 1  (50.0%) | 0  (0.0%) | 0  (0.0%) | 0  (0.0%) | 0  (0.0%) | 2  (1.5%) | 2 (100.0%) | 0  (0.0%) |
| **Total** | **133 (100.0%)** | **2**  **(1.5%)** | **16 (12.0%)** | **32**  **(24.1%)** | **33**  **(24.8%)** | **25**  **(18.8%)** | **25**  **(18.8%)** | **134 (100.0%)** | **11 (8.2%)** | **123**  **(91.8%)** |

^i^ Number of participants

#### Supplement table S9: Distribution of continuous metabolic indicators by phenotype risk group among public primary school teachers in mid-2019 in Gqeberha, South Africa

|  | **Waist circumference (cm)^i^** | | | **Triglycerides**  **(mmol/L)** | | | **High density lipoprotein cholesterol (mmol/L)** | | | **Systolic blood pressure (mmHg)** | | | **Diastolic blood pressure (mmHg)** | | | **Glycated haemoglobin (HbA1c; %)** | | |
| --- | --- | --- | --- | --- | --- | --- | --- | --- | --- | --- | --- | --- | --- | --- | --- | --- | --- | --- |
| **Phenotype** | **n^ii^ (%)** | **Med^iii^**  **(IQR^iv^)** | **M^v^**  **(SD^vi^)** | **n (%)** | **Med**  **(IQR)** | **M**  **(SD)** | **n (%)** | **Med**  **(IQR)** | **M**  **(SD)** | **n (%)** | **Med**  **(IQR)** | **M**  **(SD)** | **n (%)** | **Med**  **(IQR)** | **M**  **(SD)** | **n (%)** | **Med**  **(IQR)** | **M**  **(SD)** |
| Bone-fat-lean risk | 8  (6.0%) | 95.2 (79.3–100.5) | 92.3 (18.1) | 7  (5.4%) | 2.2  (1.9–3.2) | 2.6  (1.2) | 7  (5.4%) | 1.4  (0.8–2.0) | 1.4  (0.7) | 8  (6.2%) | 137.0 (124.8–147.5) | 137.1 (21.0) | 8  (6.2%) | 89.0 (80.0–92.2) | 85.6 (9.4) | 8  (6.1%) | 5.5  (5.3–6.0) | 6.5  (2.7) |
| Fat-lean risk | 10 (7.5%) | 97.7 (70.7–112.3) | 94.3 (23.1) | 9  (6.9%) | 2.2  (1.6–2.4) | 2.2  (1.0) | 10 (7.8%) | 1.2  (1.2–1.4) | 1.3  (0.2) | 10 (7.7%) | 129.5 (111.0–142.8) | 126.5 (19.1) | 10 (7.7%) | 79.0 (73.2–95.5) | 83.1 (13.4) | 10 (7.6%) | 5.8  (5.4–6.2) | 6.3  (1.4) |
| Fat mass risk | 76 (56.7%) | 99.7 (88.7–108.0) | 99.3 (14.7) | 74 (56.9%) | 1.5  (1.2–2.0) | 1.8  (1.1) | 72 (55.8%) | 1.3  (1.1–1.6) | 1.4  (0.3) | 74 (56.9%) | 122.0 (112.2–135.0) | 125.1 (16.0) | 74 (56.9%) | 84.0 (77.0–91.8) | 83.7 (9.7) | 75 (56.8%) | 5.5  (5.3–5.8) | 5.8  (1.0) |
| Bone-fat risk | 29 (21.6%) | 95.3 (85.2–100.2) | 93.9 (11.9) | 29 (22.3%) | 1.6  (1.1–2.4) | 1.9  (1.1) | 29 (22.5%) | 1.5  (1.3–1.7) | 1.6  (0.6) | 27 (20.8%) | 128.0 (119.0–145.0) | 133.0 (21.0) | 27 (20.8%) | 84.0 (77.0–91.0) | 85.4 (14.0) | 28 (21.2%) | 5.6  (5.3–6.0) | 6.2  (1.8) |
| Non-phenotype | 6  (4.5%) | 77.3 (69.9–97.5) | 82.7 (16.5) | 6  (4.6%) | 1.1  (1.0–1.5) | 1.3  (0.6) | 6  (4.7%) | 1.2  (1.1–1.5) | 1.3  (0.4) | 6  (4.6%) | 118.0 (109.5–126.5) | 123.5 (21.6) | 6  (4.6%) | 80.5 (75.2–85.8) | 79.7 (10.5) | 6  (4.5%) | 5.3  (5.0–5.6) | 5.8  (1.5) |
| Bone mass risk | 2  (1.5%) | 71.7 (69.5–73.8) | 71.7 (6.2) | 2  (1.5%) | 1.0  (0.9–1.2) | 1.0  (0.4) | 2  (1.6%) | 1.5  (1.5–1.5) | 1.5  (0.1) | 2  (1.5%) | 106.5 (102.2–110.8) | 106.5 (12.0) | 2  (1.5%) | 69.0 (67.5–70.5) | 69.0 (4.2) | 2  (1.5%) | 5.6  (5.5–5.6) | 5.6  (0.2) |
| Bone-lean risk | 1  (0.7%) | 64.8 (64.8–64.8) | 64.8  (—) | 1  (0.8%) | 2.2  (2.2–2.2) | 2.2  (—) | 1  (0.8%) | 1.8  (1.8–1.8) | 1.8  (—) | 1  (0.8%) | 133.0 (133.0–133.0) | 133.0  (—) | 1  (0.8%) | 92.0 (92.0–92.0) | 92.0  (—) | 1  (0.8%) | 4.8  (4.8–4.8) | 4.8  (—) |
| Lean mass risk | 2  (1.5%) | 65.5 (64.5–66.5) | 65.5 (2.8) | 2  (1.5%) | 1.2  (1.0–1.3) | 1.2  (0.3) | 2  (1.6%) | 1.8  (1.7–1.8) | 1.8  (0.1) | 2  (1.5%) | 108.5 (100.8–116.2) | 108.5 (21.9) | 2  (1.5%) | 70.5 (66.8–74.2) | 70.5 (10.6) | 2  (1.5%) | 5.3  (5.2–5.4) | 5.3  (0.1) |
| **Total** | **134 (100.0%)** | **97.6 (83.6–106.0)** | **95.5 (16.2)** | **130 (100.0%)** | **1.6**  **(1.1–2.2)** | **1.8**  **(1.1)** | **129 (100.0%)** | **1.4**  **(1.2–1.6)** | **1.4**  **(0.4)** | **130 (100.0%)** | **124.0 (113.2–137.0)** | **127.0 (18.3)** | **130 (100.0%)** | **83.5 (76.0–92.0)** | **83.6 (11.1)** | **132 (100.0%)** | **5.5**  **(5.3–5.9)** | **5.9**  **(1.4)** |

^i^ Imputed waist circumference values included

^ii^ Number of participants

^iii^ Median

^iv^ Interquartile range

^v^ Mean

^vi^ Standard deviation

#### Supplement table S10: Distribution of categorical metabolic indicators by phenotype risk group among public primary school teachers in mid-2019 in Gqeberha, South Africa

|  | **Central obesity^ii^** | | | **High triglycerides^iii^** | | | **Low high density lipoprotein cholesterol^iv^** | | | **Hypertension^v^** | | | **Hyperglycaemia^vi^** | | | **Metabolic syndrome^vii^** | | | **Metabolic syndrome component count** | | | | | | |
| --- | --- | --- | --- | --- | --- | --- | --- | --- | --- | --- | --- | --- | --- | --- | --- | --- | --- | --- | --- | --- | --- | --- | --- | --- | --- |
| **Phenotype** | **n^i^ (%)** | **No** | **Yes** | **n (%)** | **No** | **Yes** | **n (%)** | **No** | **Yes** | **n (%)** | **No** | **Yes** | **n (%)** | **No** | **Yes** | **n (%)** | **No** | **Yes** | **n (%)** | **0** | **1** | **2** | **3** | **4** | **5** |
| Bone-fat-lean risk | 8  (6.0%) | 3 (37.5%) | 5 (62.5%) | 7  (5.4%) | 1 (14.3%) | 6 (85.7%) | 7  (5.4%) | 4 (57.1%) | 3 (42.9%) | 8  (6.2%) | 2 (25.0%) | 6 (75.0%) | 8  (6.0%) | 5 (62.5%) | 3 (37.5%) | 8  (6.2%) | 3 (37.5%) | 5 (62.5%) | 6  (4.8%) | 0  (0.0%) | 1 (16.7%) | 1 (16.7%) | 2 (33.3%) | 1 (16.7%) | 1 (16.7%) |
| Fat-lean risk | 10 (7.5%) | 4 (40.0%) | 6 (60.0%) | 9  (6.9%) | 4 (44.4%) | 5 (55.6%) | 10 (7.8%) | 7 (70.0%) | 3 (30.0%) | 10 (7.7%) | 5 (50.0%) | 5 (50.0%) | 10 (7.5%) | 5 (50.0%) | 5 (50.0%) | 10 (7.7%) | 5 (50.0%) | 5 (50.0%) | 9  (7.3%) | 2 (22.2%) | 0  (0.0%) | 2 (22.2%) | 1 (11.1%) | 3 (33.3%) | 1 (11.1%) |
| Fat mass risk | 76 (56.7%) | 9 (11.8%) | 67 (88.2%) | 74 (56.9%) | 41 (55.4%) | 33 (44.6%) | 72 (55.8%) | 40 (55.6%) | 32 (44.4%) | 74 (56.9%) | 33 (44.6%) | 41 (55.4%) | 76 (57.1%) | 48 (63.2%) | 28 (36.8%) | 73 (56.2%) | 32 (43.8%) | 41 (56.2%) | 71 (57.3%) | 3  (4.2%) | 14 (19.7%) | 15 (21.1%) | 16 (22.5%) | 17 (23.9%) | 6  (8.5%) |
| Bone-fat risk | 29 (21.6%) | 4 (13.8%) | 25 (86.2%) | 29 (22.3%) | 16 (55.2%) | 13 (44.8%) | 29 (22.5%) | 22 (75.9%) | 7 (24.1%) | 27 (20.8%) | 10 (37.0%) | 17 (63.0%) | 28 (21.1%) | 15 (53.6%) | 13 (46.4%) | 28 (21.5%) | 13 (46.4%) | 15 (53.6%) | 27 (21.8%) | 1  (3.7%) | 4 (14.8%) | 8 (29.6%) | 7 (25.9%) | 6 (22.2%) | 1  (3.7%) |
| Non-phenotype | 6  (4.5%) | 4 (66.7%) | 2 (33.3%) | 6  (4.6%) | 5 (83.3%) | 1 (16.7%) | 6  (4.7%) | 4 (66.7%) | 2 (33.3%) | 6  (4.6%) | 4 (66.7%) | 2 (33.3%) | 6  (4.5%) | 5 (83.3%) | 1 (16.7%) | 6  (4.6%) | 4 (66.7%) | 2 (33.3%) | 6  (4.8%) | 2 (33.3%) | 2 (33.3%) | 0  (0.0%) | 2 (33.3%) | 0  (0.0%) | 0  (0.0%) |
| Bone mass risk | 2  (1.5%) | 2 (100%) | 0  (0.0%) | 2  (1.5%) | 2 (100%) | 0  (0.0%) | 2  (1.6%) | 2 (100%) | 0  (0.0%) | 2  (1.5%) | 2 (100%) | 0  (0.0%) | 2  (1.5%) | 1 (50.0%) | 1 (50.0%) | 2  (1.5%) | 2 (100%) | 0  (0.0%) | 2  (1.6%) | 1 (50.0%) | 1 (50.0%) | 0  (0.0%) | 0  (0.0%) | 0  (0.0%) | 0  (0.0%) |
| Bone-lean risk | 1  (0.7%) | 1 (100%) | 0  (0.0%) | 1  (0.8%) | 0  (0.0%) | 1 (100%) | 1  (0.8%) | 1 (100%) | 0  (0.0%) | 1  (0.8%) | 0  (0.0%) | 1 (100%) | 1  (0.8%) | 1 (100%) | 0  (0.0%) | 1  (0.8%) | 1 (100%) | 0  (0.0%) | 1  (0.8%) | 0  (0.0%) | 0  (0.0%) | 1 (100%) | 0  (0.0%) | 0  (0.0%) | 0  (0.0%) |
| Lean mass risk | 2  (1.5%) | 2 (100%) | 0  (0.0%) | 2  (1.5%) | 2 (100%) | 0  (0.0%) | 2  (1.6%) | 2 (100%) | 0  (0.0%) | 2  (1.5%) | 2 (100%) | 0  (0.0%) | 2  (1.5%) | 2 (100%) | 0  (0.0%) | 2  (1.5%) | 2 (100%) | 0  (0.0%) | 2  (1.6%) | 2 (100%) | 0  (0.0%) | 0  (0.0%) | 0  (0.0%) | 0  (0.0%) | 0  (0.0%) |
| **Total** | **134 (100%)** | **29 (21.6%)** | **105 (78.4%)** | **130 (100%)** | **71 (54.6%)** | **59 (45.4%)** | **129 (100%)** | **82 (63.6%)** | **47 (36.4%)** | **130 (100%)** | **58 (44.6%)** | **72 (55.4%)** | **133 (100%)** | **82 (61.7%)** | **51 (38.3%)** | **130 (100%)** | **62 (47.7%)** | **68 (52.3%)** | **124 (100%)** | **11 (8.9%)** | **22 (17.7%)** | **27 (21.8%)** | **28 (22.6%)** | **27 (21.8%)** | **9**  **(7.3%)** |

^i^ Number of participants

^ii^ Population-specific waist circumference: ≥80 cm for women and ≥94 cm for men in sub-Saharan African, European, Middle Eastern, and Mediterranean populations.

^iii^ Fasting triglyceride levels: ≥1.7 mmol/L or on treatment for dyslipidaemia.

^iv^ High-density lipoprotein cholesterol: <1.3 mmol/L in women and <1.0 mmol/L in men or on treatment for dyslipidaemia.

^v^ Systolic blood pressure ≥130 mmHg and/or diastolic blood pressure: ≥85 mmHg or on treatment for hypertension.

^vi^ Fasting glucose: ≥5.6 mmol/L, equivalent to glycated haemoglobin (HbA1c) ≥5.7% or on treatment for hyperglycaemia.

^vii^ Metabolic syndrome if 3/5 criteria are present (central obesity, high triglycerides, low HDL-C, hypertension, and/or hyperglycaemia).

#### Supplement table S11: Distribution of categorical substance use indicators by phenotype risk group among public primary school teachers in mid-2019 in Gqeberha, South Africa

|  | **Tobacco use** | | | **Alcohol consumption** | | |
| --- | --- | --- | --- | --- | --- | --- |
| **Phenotype** | **n^i^ (%)** | **No** | **Yes** | **n (%)** | **No** | **Yes** |
| Bone-fat-lean risk | 8  (6.0%) | 8  (100.0%) | 0  (0.0%) | 8  (6.0%) | 5  (62.5%) | 3  (37.5%) |
| Fat-lean risk | 10  (7.5%) | 8  (80.0%) | 2  (20.0%) | 10  (7.5%) | 5  (50.0%) | 5  (50.0%) |
| Fat mass risk | 76  (56.7%) | 62  (81.6%) | 14  (18.4%) | 76  (56.7%) | 46  (60.5%) | 30  (39.5%) |
| Bone-fat risk | 29  (21.6%) | 26  (89.7%) | 3  (10.3%) | 29  (21.6%) | 25  (86.2%) | 4  (13.8%) |
| Non-phenotype | 6  (4.5%) | 4  (66.7%) | 2  (33.3%) | 6  (4.5%) | 2  (33.3%) | 4  (66.7%) |
| Bone mass risk | 2  (1.5%) | 2  (100.0%) | 0  (0.0%) | 2  (1.5%) | 2  (100.0%) | 0  (0.0%) |
| Bone-lean risk | 1  (0.7%) | 0  (0.0%) | 1  (100.0%) | 1  (0.7%) | 0  (0.0%) | 1  (100.0%) |
| Lean mass risk | 2  (1.5%) | 1  (50.0%) | 1  (50.0%) | 2  (1.5%) | 1  (50.0%) | 1  (50.0%) |
| **Total** | **134 (100.0%)** | **111**  **(82.8%)** | **23**  **(17.2%)** | **134 (100.0%)** | **86**  **(64.2%)** | **48**  **(35.8%)** |

^i^ Number of participants

#### Supplement table S12: Distribution of continuous physical activity indicators by phenotype risk group among public primary school teachers in mid-2019 in Gqeberha, South Africa

|  | **Euclidean Norm Minus One; negative values set to zero (m*g*/day)^i^** | | | **Moderate-to-vigorous intensity physical activity (min/week)** | | | **Absolute maximum grip strength**  **(kg)** | | |
| --- | --- | --- | --- | --- | --- | --- | --- | --- | --- |
| **Phenotype** | **n^ii^ (%)** | **Med^iii^**  **(IQR^iv^)** | **M^v^**  **(SD^vi^)** | **n (%)** | **Med**  **(IQR)** | **M**  **(SD)** | **n (%)** | **Med**  **(IQR)** | **M**  **(SD)** |
| Bone-fat-lean risk | 3  (4.0%) | 13.7  (12.6–14.5) | 13.5  (1.9) | 3  (4.0%) | 83.7  (62.4–  86.0) | 71.0  (26.1) | 8  (6.0%) | 26.5  (23.0–33.0) | 28.4  (6.5) |
| Fat-lean risk | 8  (10.7%) | 13.2  (11.0–14.6) | 12.9  (3.1) | 8  (10.7%) | 162.5  (124.1–175.6) | 152.0  (68.9) | 10  (7.5%) | 25.0  (24.0–34.5) | 29.1  (7.5) |
| Fat mass risk | 37  (49.3%) | 13.8  (10.1–16.1) | 13.6  (4.5) | 37  (49.3%) | 119.0 (77.5–200.3) | 140.6  (90.0) | 76  (56.7%) | 34.5  (31.0–40.0) | 36.0  (6.1) |
| Bone-fat risk | 21  (28.0%) | 13.0  (8.7–14.6) | 12.4  (4.3) | 21  (28.0%) | 115.0 (88.3–164.2) | 135.9  (82.1) | 29  (21.6%) | 34.0  (29.0–36.0) | 32.9  (5.1) |
| Non-phenotype | 3  (4.0%) | 12.9  (10.3–17.5) | 14.2  (7.3) | 3  (4.0%) | 42.7  (38.1–233.0) | 166.5 (222.4) | 6  (4.5%) | 40.0  (35.2–47.0) | 43.0  (13.1) |
| Bone mass risk | 1  (1.3%) | 15.7  (15.7–15.7) | 15.7  (—) | 1  (1.3%) | 175.0 (175.0–175.0) | 175.0  (—) | 2  (1.5%) | 38.0  (33.0–43.0) | 38.0  (14.1) |
| Bone-lean risk | 0  (0.0%) | — | — | 0  (0.0%) | — | — | 1  (0.7%) | 40.0  (40.0–40.0) | 40.0  (—) |
| Lean mass risk | 2  (2.7%) | 25.2  (24.0–26.4) | 25.2  (3.5) | 2  (2.7%) | 496.4 (439.1–553.7) | 496.4 (162.1) | 2  (1.5%) | 32.5  (31.8–33.2) | 32.5  (2.1) |
| **Total** | **75**  **(100.0%)** | **13.4**  **(10.2–16.0)** | **13.6**  **(4.7)** | **75**  **(100.0%)** | **124.0 (76.1–198.9)** | **148.7 (107.4)** | **134 (100.0%)** | **34.0**  **(30.0–39.0)** | **34.7**  **(7.1)** |

^i^ m*g:* milligravity

^ii^ Number of participants

^iii^ Median

^iv^ Interquartile range

^v^ Mean

^vi^ Standard deviation

#### Supplement table S13: Distribution of categorical physical activity indicators by phenotype risk group among public primary school teachers in mid-2019 in Gqeberha, South Africa

|  | **Met WHO physical activity guideline^i^** | | | **Absolute maximum grip strength classification** | | | | | **Weight bearing exercise^ii^** | | |
| --- | --- | --- | --- | --- | --- | --- | --- | --- | --- | --- | --- |
| **Phenotype** | **n^iii^ (%)** | **No** | **Yes** | **n (%)** | **Low** | **Average** | **Good** | **Excellent** | **n (%)** | **No** | **Yes** |
| Bone-fat-lean risk | 3  (4.0%) | 3 (100.0%) | 0  (0.0%) | 8  (6.0%) | 7  (87.5%) | 1  (12.5%) | 0  (0.0%) | 0  (0.0%) | 7  (7.0%) | 5 (71.4%) | 2 (28.6%) |
| Fat-lean risk | 8  (10.7%) | 3 (37.5%) | 5 (62.5%) | 10 (7.5%) | 9  (90.0%) | 1  (10.0%) | 0  (0.0%) | 0  (0.0%) | 5  (5.0%) | 4 (80.0%) | 1 (20.0%) |
| Fat mass risk | 37  (49.3%) | 22 (59.5%) | 15 (40.5%) | 76 (56.7%) | 0  (0.0%) | 46 (60.5%) | 12  (15.8%) | 18  (23.7%) | 57 (57.0%) | 36 (63.2%) | 21 (36.8%) |
| Bone-fat risk | 21  (28.0%) | 14 (66.7%) | 7 (33.3%) | 29 (21.6%) | 0  (0.0%) | 18 (62.1%) | 6  (20.7%) | 5  (17.2%) | 21 (21.0%) | 16 (76.2%) | 5 (23.8%) |
| Non-phenotype | 3  (4.0%) | 2 (66.7%) | 1 (33.3%) | 6  (4.5%) | 0  (0.0%) | 3  (50.0%) | 1  (16.7%) | 2  (33.3%) | 6  (6.0%) | 2 (33.3%) | 4 (66.7%) |
| Bone mass risk | 1  (1.3%) | 0  (0.0%) | 1 (100.0%) | 2  (1.5%) | 0  (0.0%) | 2 (100.0%) | 0  (0.0%) | 0  (0.0%) | 2  (2.0%) | 1 (50.0%) | 1 (50.0%) |
| Bone-lean risk | 0  (0.0%) | 0  (0.0%) | 0  (0.0%) | 1  (0.7%) | 1  (100.0%) | 0  (0.0%) | 0  (0.0%) | 0  (0.0%) | 0  (0.0%) | 0 (0.0%) | 0  (0.0%) |
| Lean mass risk | 2  (2.7%) | 0  (0.0%) | 2 (100.0%) | 2  (1.5%) | 1  (50.0%) | 1  (50.0%) | 0  (0.0%) | 0  (0.0%) | 2  (2.0%) | 0 (0.0%) | 2 (100.0%) |
| **Total** | **75**  **(100.0%)** | **44 (58.7%)** | **31 (41.3%)** | **134 (100.0%)** | **18**  **(13.4%)** | **72 (53.7%)** | **19**  **(14.2%)** | **25**  **(18.7%)** | **100 (100.0%)** | **64 (64.0%)** | **36 (36.0%)** |

^i^ Met World Health Organization (WHO) physical activity guideline of a minimum of 150 mins of moderate-to-vigorous intensity physical activity (MVPA) per week.

^ii^ “Are you currently participating in regular weight-bearing exercise?”

^iii^ Number of participants

#### Supplement table S14: Distribution of categorical medical history indicators by phenotype risk group among public primary school teachers in mid-2019 in Gqeberha, South Africa

|  | **Diagnosis of secondary osteoporosis** | | | **Treatment for osteoporosis** | | | **Treatment for dyslipidaemia** | | | **Treatment for hypertension** | | | **Treatment for hyperglycaemia** | | |
| --- | --- | --- | --- | --- | --- | --- | --- | --- | --- | --- | --- | --- | --- | --- | --- |
| **Phenotype** | **n^i^ (%)** | **No** | **Yes** | **n (%)** | **No** | **Yes** | **n (%)** | **No** | **Yes** | **n (%)** | **No** | **Yes** | **n (%)** | **No** | **Yes** |
| Bone-fat-lean risk | 7  (6.2%) | 7 (100.0%) | 0  (0.0%) | 7  (6.2%) | 7 (100.0%) | 0  (0.0%) | 8  (6.0%) | 8 (100.0%) | 0 (0.0%) | 8  (6.0%) | 6 (75.0%) | 2  (25.0%) | 8  (6.0%) | 6 (75.0%) | 2  (25.0%) |
| Fat-lean risk | 6  (5.4%) | 6 (100.0%) | 0  (0.0%) | 6  (5.4%) | 6 (100.0%) | 0  (0.0%) | 10 (7.5%) | 8  (80.0%) | 2 (20.0%) | 10 (7.5%) | 8 (80.0%) | 2  (20.0%) | 10 (7.5%) | 8 (80.0%) | 2  (20.0%) |
| Fat mass risk | 63 (56.2%) | 63 (100.0%) | 0  (0.0%) | 63 (56.2%) | 63 (100.0%) | 0  (0.0%) | 76 (56.7%) | 68 (89.5%) | 8 (10.5%) | 76 (56.7%) | 64 (84.2%) | 12 (15.8%) | 76 (56.7%) | 72 (94.7%) | 4  (5.3%) |
| Bone-fat risk | 25 (22.3%) | 25 (100.0%) | 0  (0.0%) | 25 (22.3%) | 25 (100.0%) | 0  (0.0%) | 29 (21.6%) | 25 (86.2%) | 4 (13.8%) | 29 (21.6%) | 25 (86.2%) | 4  (13.8%) | 29 (21.6%) | 25 (86.2%) | 4  (13.8%) |
| Non-phenotype | 6  (5.4%) | 6 (100.0%) | 0  (0.0%) | 6  (5.4%) | 6 (100.0%) | 0  (0.0%) | 6  (4.5%) | 6 (100.0%) | 0 (0.0%) | 6  (4.5%) | 6 (100.0%) | 0  (0.0%) | 6  (4.5%) | 5 (83.3%) | 1  (16.7%) |
| Bone mass risk | 2  (1.8%) | 2 (100.0%) | 0  (0.0%) | 2  (1.8%) | 2 (100.0%) | 0  (0.0%) | 2  (1.5%) | 2 (100.0%) | 0 (0.0%) | 2  (1.5%) | 2 (100.0%) | 0  (0.0%) | 2  (1.5%) | 2 (100.0%) | 0  (0.0%) |
| Bone-lean risk | 1  (0.9%) | 1 (100.0%) | 0  (0.0%) | 1  (0.9%) | 1 (100.0%) | 0  (0.0%) | 1  (0.7%) | 1 (100.0%) | 0 (0.0%) | 1  (0.7%) | 1 (100.0%) | 0  (0.0%) | 1  (0.7%) | 1 (100.0%) | 0  (0.0%) |
| Lean mass risk | 2  (1.8%) | 2 (100.0%) | 0  (0.0%) | 2  (1.8%) | 2 (100.0%) | 0  (0.0%) | 2  (1.5%) | 2 (100.0%) | 0 (0.0%) | 2  (1.5%) | 2 (100.0%) | 0  (0.0%) | 2  (1.5%) | 2 (100.0%) | 0  (0.0%) |
| **Total** | **112 (100.0%)** | **112 (100.0%)** | **0**  **(0.0%)** | **112 (100.0%)** | **112 (100.0%)** | **0**  **(0.0%)** | **134 (100.0%)** | **120 (89.6%)** | **14 (10.4%)** | **134 (100.0%)** | **114 (85.1%)** | **20 (14.9%)** | **134 (100.0%)** | **121 (90.3%)** | **13**  **(9.7%)** |

^i^ Number of participants
